# Colonic Butyrate-Producing Communities in Humans: an Overview Using Omics Data

**DOI:** 10.1128/mSystems.00130-17

**Published:** 2017-12-05

**Authors:** Marius Vital, André Karch, Dietmar H. Pieper

**Affiliations:** a Microbial Interactions and Processes Research Group, Helmholtz Centre for Infection Research, Braunschweig, Germany; b Epidemiological and Statistical Methods Research Group, Helmholtz Centre for Infection Research, Braunschweig, Germany; Michigan State University

**Keywords:** butyrate, cardiometabolic disease, ecology, functional stability, gut microbiota

## Abstract

Given the key role of butyrate for host health, understanding the ecology of intestinal butyrate-producing communities is a top priority for gut microbiota research. To this end, we performed a pooled analysis on 2,387 metagenomic/transcriptomic samples from 15 publicly available data sets that originated from three continents and encompassed eight diseases as well as specific interventions. For analyses, a gene catalogue was constructed from gene-targeted assemblies of all genes from butyrate synthesis pathways of all samples and from an updated reference database derived from genome screenings. We demonstrate that butyrate producers establish themselves within the first year of life and display high abundances (>20% of total bacterial community) in adults regardless of origin. Various bacteria form this functional group, exhibiting a biochemical diversity including different pathways and terminal enzymes, where one carbohydrate-fueled pathway was dominant with butyryl coenzyme A (CoA):acetate CoA transferase as the main terminal enzyme. Subjects displayed a high richness of butyrate producers, and 17 taxa, primarily members of the *Lachnospiraceae* and *Ruminococcaceae* along with some *Bacteroidetes*, were detected in >70% of individuals, encompassing ~85% of the total butyrate-producing potential. Most of these key taxa were also found to express genes for butyrate formation, indicating that butyrate producers occupy various niches in the gut ecosystem, concurrently synthesizing that compound. Furthermore, results from longitudinal analyses propose that diversity supports functional stability during ordinary life disturbances and during interventions such as antibiotic treatment. A reduction of the butyrate-producing potential along with community alterations was detected in various diseases, where patients suffering from cardiometabolic disorders were particularly affected.

**IMPORTANCE** Studies focusing on taxonomic compositions of the gut microbiota are plentiful, whereas its functional capabilities are still poorly understood. Specific key functions deserve detailed investigations, as they regulate microbiota-host interactions and promote host health and disease. The production of butyrate is among the top targets since depletion of this microbe-derived metabolite is linked to several emerging noncommunicable diseases and was shown to facilitate establishment of enteric pathogens by disrupting colonization resistance. In this study, we established a workflow to investigate in detail the composition of the polyphyletic butyrate-producing community from omics data extracting its biochemical and taxonomic diversity. By combining information from various publicly available data sets, we identified universal ecological key features of this functional group and shed light on its role in health and disease. Our results will assist the development of precision medicine to combat functional dysbiosis.

## Abstract (precis)

Studies focusing on taxonomic compositions of the gut microbiota are plentiful, whereas its functional capabilities are still poorly understood. Specific key functions deserve detailed investigations, as they regulate microbiota-host interactions and promote host health and disease. The production of butyrate is among the top targets since depletion of this microbe-derived metabolite is linked to several emerging noncommunicable diseases and was shown to facilitate establishment of enteric pathogens by disrupting colonization resistance. In this study, we established a workflow to investigate in detail the composition of the polyphyletic butyrate-producing community from omics data extracting its biochemical and taxonomic diversity. By combining information from various publicly available data sets, we identified universal ecological key features of this functional group and shed light on its role in health and disease. Our results will assist the development of precision medicine to combat functional dysbiosis.

## INTRODUCTION

Butyrate, produced by the intestinal microbiota, is essential to maintaining host health by providing energy to the intestinal epithelium, modulating the immune system, and affecting diverse metabolic routes throughout the body, e.g., in the liver and the brain (1, 2). Depletion in butyrate-producing taxa has been linked to several emerging noncommunicable diseases, such as type 2 diabetes (T2D) (3), obesity (4), and cardiovascular disease (5), and was shown to facilitate establishment of enteric pathogens by disrupting colonization resistance (6). Four major pathways and several biochemically distinct enzymes that perform the terminal formation of butyrate were described, encompassing taxa from various *Firmicutes* families and some *Bacteroidetes* (7). In microbiota research, taxonomic approaches that focus on individual taxa prevail, whereas functional capabilities of entire communities are usually neglected. Even in most omics-based studies, taxonomic analyses predominate, and comprehensive analyses on butyrate-producing pathways including all associated taxa are lacking. Since the butyrate-producing community is polyphyletic and forms a biochemically diverse group, detailed taxon-function investigations are required in order to accurately characterize this functional guild.

In this study, we established a “function-centric” approach that enables detailed insights into butyrate-producing communities from omics data revealing its biochemical (different pathways and terminal enzymes) and taxonomic diversity. We aimed to investigate the role of butyrate producers in health and disease and to extract ecological key features of this functional group in order to assist the design of precision medicine that combats functional dysbiosis. To this end, 15 publicly available metagenomic and metatranscriptomic data sets (*n* = 2,387 samples) were analyzed after establishing a gene catalogue via gene-targeted assemblies considering all major known pathways, terminal enzymes, and associated taxa. The data sets derived from North America, Europe, and Asia and involved eight diseases and specific interventions as well as one study that followed bacterial community succession after birth (8). We characterized the butyrate-producing community in detail during functional dysbiosis and identified the roles of individual taxa in functional resistance and resilience during disturbances.

## RESULTS

**FIG 1:**
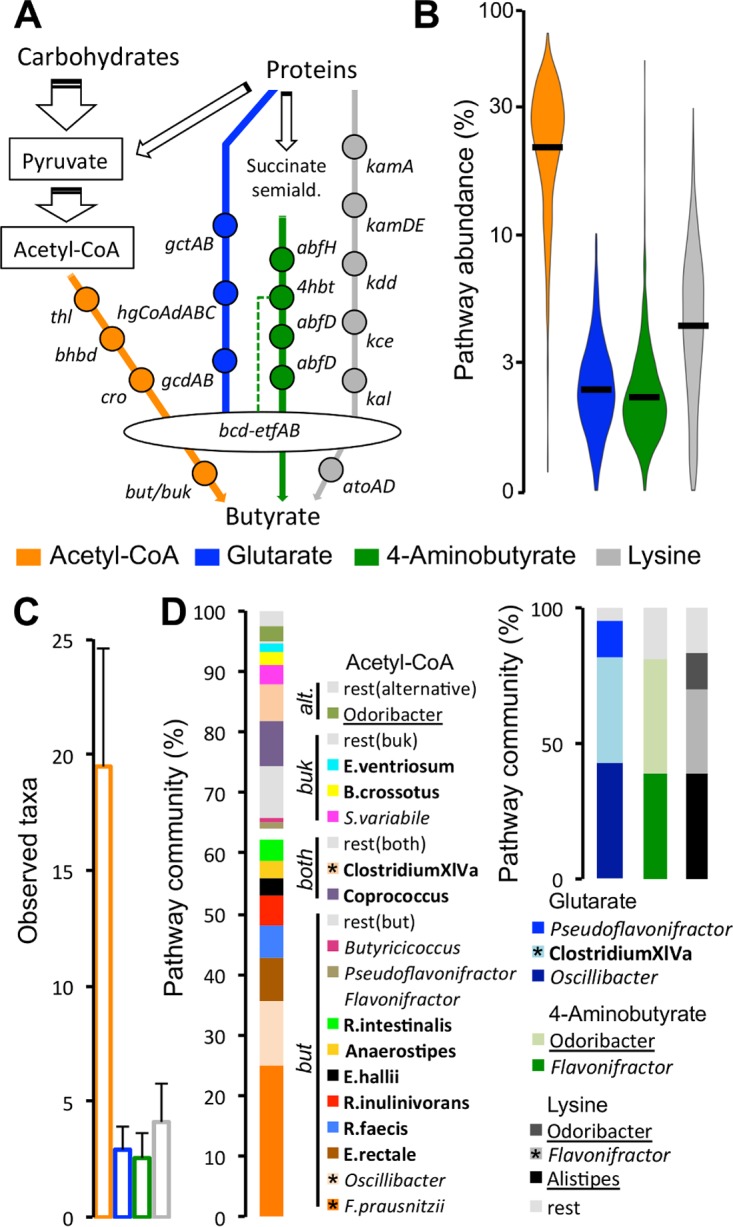
Characterization of the butyrate-producing community in samples derived from healthy individuals of nine metagenomic studies (I to IX; *n* = 826). (A) Overview of butyrate-forming pathways, including all major genes involved. (B) Abundances of bacteria exhibiting respective pathways as percentages of total bacteria. (C) Mean number of observed taxa associated with each pathway in these samples. (D) Relative abundances of taxa comprising all pathways. Only taxa that were detected in >70% of all individuals are shown (along with *Butyrivibrio crossotus*, which showed high abundances in several samples); all taxa shown are known butyrate producers. Bacteria exhibiting the acetyl-CoA pathway are arranged according to their terminal enzymes, butyryl-CoA:acetate CoA transferase (*but*) and butyrate kinase (*buk*); bacteria exhibiting *but* and *buk* (both) or lacking both enzymes (alternative [alt.]) are also indicated. Members of the *Lachnospiraceae* are indicated in bold, *Ruminococcaceae* are indicated in italics, and *Bacteroidetes* are underlined. Black bars in violin plots (B) represent means. Error bars represent standard deviations. *, taxa detected in >90% of individuals. *gct*, glutaconate-CoA transferase (α, β subunit); *hgCoAd*, 2-hydroxyglutaryl-CoA dehydratase (α, β, and γ subunit); *gcd*, glutaconyl-CoA decarboxylase (α, β subunit); *thl*, acetyl-CoA acetyltransferase (thiolase); *bhbd*, β-hydroxybutyryl-CoA dehydrogenase; *cro*, crotonase; *bcd*, butyryl-CoA dehydrogenase (including electron transfer protein α, β subunit); *kamA*, lysine-2,3-aminomutase; *kamDE*, β-lysine-5,6-aminomutase (α, β subunit); *kdd*, 3,5-diaminohexanoate dehydrogenase; *kce*, 3-keto-5-aminohexanoate cleavage enzyme; *kal*, 3-aminobutyryl-CoA ammonia-lyase; *abfH*, 4-hydroxybutyrate dehydrogenase; *abfD*, 4-hydroxybutyryl-CoA dehydratase and vinylacetyl-CoA 3,2-isomerase (same gene); *4hbt*, butyryl-CoA:4-hydroxybutyrate CoA transferase; *ato*, butyryl-CoA:acetoacetate CoA transferase (α, β subunit).

An overview of all pathways and genes involved in the formation of butyrate is presented in Fig. 1A. The acetyl coenzyme A (CoA) pathway (Ac pathway) represents the main pathway that is fueled by carbohydrates, the major energy source for colonic bacteria, whereas the glutarate (Gl), 4-aminobutyrate (4A), and lysine (Ly) pathways are fed by proteins (7, 9). In this study, we constructed a butyrate-specific gene catalogue via gene-targeted assemblies of all pathway genes from 15 omics data sets in order to quantify community members of the colonic microbiota that harbor the respective pathways and determine their taxonomic composition. An overview of the individual studies is given in Table 1. For simplicity, we refer to them as studies I to XV throughout this work. Since taxon abundances based on results of respective pathway genes correlated well with abundances of corresponding housekeeping genes (Spearman’s ρ > 0.8 for most taxa; see Table S1B and C in the supplemental material), taxonomic affiliations of pathway genes will be used to describe results throughout the text.

10.1128/mSystems.00130-17.9 TABLE S1  Sheet A gives an overview of individual data sets included in our analyses. All metagenomic (metaG) and metatranscriptomic (metaT) data derived from fecal samples. The genome name, taxonomic classification based on RDP taxonomy on the genus level, and taxonomic affiliation used in the study are given for all butyrate-producing candidates in sheet B. In sheet C, correlations (Spearman’s rho) between mean abundances of pathway genes and mean abundances of the corresponding three housekeeping genes (hkg) *rplB/recA/pyrG* for all major taxa considering all samples included in this study (*n* = 2,387) are shown. Download TABLE S1, XLSX file, 0.1 MB. Copyright © 2017 Vital et al. 2017 Vital et al. This content is distributed under the terms of the Creative Commons Attribution 4.0 International license.

**TABLE 1:** Overview of individual data sets included in this study^a^

Study	Reference	Short description	Data type
I	HMP, 2012 (10)	Samples of healthy individuals (*n* = 154)	MG
II	Vogtmann et al., 2016 (11)	CRC (*n* = 52) vs controls (*n* = 52)	MG
IIIa	Le Chatelier et al*.*, 2013 (4)	Obese (*n* = 169) vs controls (*n* = 123)	MG
IIIb	Qin et al., 2010 (19)	UC (*n* = 21), CD (*n* = 4) vs controls (*n* = 14)	MG
IV	Karlsson et al., 2013 (12)	T2D (*n* = 53) vs controls (*n* = 49)	MG
V	Zeller et al., 2014 (13)	CRC (*n* = 91) vs controls (*n* = 66)	MG
VI	Bäckhed et al., 2015 (8)	Mothers (*n* = 100) and their infants, 1 wk (*n* = 98), 4 and 12 mo (*n* = 100 each)	MG
VII	Karlsson et al., 2012 (5)	CVD (*n* = 13) vs controls (*n* = 12)	MG
VIII	Qin et al., 2012 (3)	T2D (*n* = 182) vs controls (*n* = 185)	MG
IX	Qin et al., 2014 (14)	Cirrhosis (*n* = 123) vs controls (*n* = 114)	MG
X	Franzosa et al., 2014 (15)	MG and MT of 8 individuals	MG/MT
XI	David et al., 2013 (16)	10 subjects receiving plant-based and animal-product-based diets	MT
XII	Forslund et al., 2015 (18)	T2D (*n* = 75), T1D (*n* = 31) vs controls from III	MG
XIII	Raymond et al., 2015 (20)	Antibiotic treatment (*n* = 18) vs controls (*n* = 6)	MG
XIV	Zhang et al., 2015 (21)	Dietary intervention in simple (diet-related, *n* = 21) and genetic (*n* = 17) obesity	MG
XV	Li et al., 2016 (22)	Fecal transplantation (*n* = 5) vs placebo (*n* = 5)	MG

a For more details, see Table S1. Abbreviations: CRC, colorectal cancer; UC, ulcerative colitis; CD, Crohn’s disease; T1(2)D, type 1 (type 2) diabetes; CVD, cardiovascular disease; MG, metagenomic data; MT, metatranscriptomic data; HMP, Human Microbiome Project.

### Abundance and composition of the colonic butyrate-producing community.

In order to gain a global overview of colonic bacteria exhibiting butyrate-producing pathways, we analyzed metagenomes derived from three continents—North America (studies I and II [10, 11]), Europe (studies III to VII [4, 5, 8, 12, 13]), and Asia (studies VIII and IX [3, 14])—considering only samples from individuals without known disease (“healthy”; for study VI, only samples from mothers were considered) (*n* = 826).

The mean abundance of the dominant Ac pathway was high: 24.2% ± 11.3% of bacteria of the total community exhibited this pathway, with almost all samples (95.8%) displaying abundances of >5% (Fig. 1B). Other pathways displayed lower mean abundances, namely, 1.8% ± 1.3% (Gl), 1.7% ± 2.4% (4A), and 4.4% ± 3.7% (Ly), with 70.3% (Gl), 61.4% (4A), and 85.7% (Ly) of samples exhibiting communities where >1% of bacteria harbored respective pathways (Fig. 1B). On average, 19.5 ± 5.1 taxa were contributing to the Ac pathway, whereas fewer taxa were detected harboring other pathways (2.9 ± 1.0 [Gl], 2.6 ± 1.1 [4A], and 4.1 ± 1.7 [Ly]) (Fig. 1C). For the Ac pathway, 16 taxa that were detected in >70% of individuals accounted for 86.9% ± 13.5% of the total pathway community (Fig. 1D); all are known butyrate producers. Bacteria showing butyrate transferase (*but*) as the terminal enzyme were dominant (74.3% ± 13.7%) over butyrate kinase (*buk*)-exhibiting bacteria (7.1% ± 8.8%). Taxa containing both *but* and *buk* (13.4% ± 9.1%) as well as bacteria lacking these genes (5.1% ± 7.8%; referred to as “alternative” below) were observed as well (Fig. 1D). The majority of taxa were associated with the *Firmicutes* families *Lachnospiraceae* (47.8% ± 18.4%) and *Ruminococcaceae* (43.2% ± 17.6%), whereas *Porphyromonadaceae* (*Bacteroidetes*) (3.8% ± 6.2%) and other taxa (5.2% ± 11.8%; referred to as “others” below) were observed to a minor extent (Fig. 1D). Three taxa, namely, *Faecalibacterium prausnitzii*, *Oscillibacter*, and *Clostridium* XIVa, were detected in >90% of all samples, forming a global core community. They constituted 41.7% ± 15.9% of all bacteria associated with the Ac pathway. Three (Gl and Ly) and two (4A) specific taxa associated with protein-fed pathways were present in >70% of samples, representing 94.8% ± 12.3% (Gl), 80.7% ± 22.5% (4A), and 83.1% ± 20.4% (Ly) of respective communities (Fig. 1D); all are known butyrate producers and additionally exhibit the Ac pathway, except for *Alistipes*, which contained the Ly pathway as the only route for butyrate synthesis. *Clostridium* XIVa (Gl) and *Flavonifractor* (Ly) were detected in >90% of samples (Fig. 1D).

Overall, mean pathway abundances were similar in all data sets, except for the Human Microbiome Project (HMP) (study I), which displayed lower Ac pathway levels (Fig. S1A). The same taxa were dominant in all data sets (Fig. S1C to F); nevertheless, compositions of the butyrate-producing communities differed between individuals originating from distinct continents (Fig. S2). For instance, Europeans showed increased relative abundances of *Anaerostipes*, *Coprococcus*, *Eubacterium hallii*, and *Subdoligranulum variabile*, whereas levels of *Clostridium* XIVa and *Roseburia inulinivorans* were elevated in Chinese individuals (Fig. S2B). *Oscillibacter* and several *Bacteroidetes* were increased in United States-derived samples (mainly in the HMP data set).

10.1128/mSystems.00130-17.1 FIG S1  Characterization of the butyrate-producing community in samples derived from healthy individuals encompassing nine metagenomic (studies I to IX) and two metatranscriptomic (X and XI) data sets. Panel A displays the abundances of pathways as percentages of total bacteria (relative to the mean abundance of three housekeeping genes; for metatranscriptomes, mean expression of the same genes was used as the reference). The mean number of observed taxa associated with each pathway is shown in panel B. Panels C to F show relative abundances of taxa encoding/expressing respective pathways. Only taxa that were detected in >70% of all samples (*n* = 826) are displayed (along with *Butyrivibrio crossotus*, which showed high abundances in several samples); all taxa shown are known butyrate producers. Bacteria exhibiting the acetyl-CoA pathway are arranged according to their terminal enzymes butyryl-CoA:acetate CoA transferase (*but*) and butyrate kinase (*buk*); bacteria exhibiting *but* and *buk* (both) or lacking both enzymes (alternative) are also indicated. Members of the *Lachnospiraceae* are indicated in bold, *Ruminococcaceae* are indicated in italics, and *Bacteroidetes* are underlined. Error bars represent standard deviations. For the key to Roman numerals referring to individual studies, see Table 1. Download FIG S1, JPG file, 0.5 MB. Copyright © 2017 Vital et al. 2017 Vital et al. This content is distributed under the terms of the Creative Commons Attribution 4.0 International license.

10.1128/mSystems.00130-17.2 FIG S2  Differences in butyrate-producing communities between continents. Panel A shows nonmetric multidimensional scaling analyses of butyrate-producing communities from individuals derived from the United States (I and II), Europe (III to VII), and China (VIII and IX). Tips of lines illustrate individual samples, whereas ellipses represent standard deviations of points. Relative abundances of acetyl-CoA pathway groups, i.e., “enzyme” (cumulative abundance of all taxa exhibiting distinct terminal enzymes) and “family” (cumulative abundance of all taxa associated with respective taxonomic families), and of individual taxa are shown in panel B. Pathway affiliations of taxa are indicated by the color bars; members of the acetyl-CoA pathway are arranged on the family level. Acetyl-CoA pathway groups and all taxa shown differed significantly between continents (*P* < 0.05 in Kruskal-Wallis test; FDR corrected). For the key to Roman numerals referring to individual studies, see Table 1. Download FIG S2, JPG file, 0.4 MB. Copyright © 2017 Vital et al. 2017 Vital et al. This content is distributed under the terms of the Creative Commons Attribution 4.0 International license.

In metatranscriptomic data (studies X [15] and XI [16]), expression of the Ac pathway was also dominant over protein-fed pathways, and genes associated with the same taxa that prevailed in metagenomes displayed the highest transcription levels (Fig. S1A to F). The richness of taxa expressing butyrate-forming pathways in study X was similar to the number of taxa expressing the respective enzymes observed in metagenomic data, whereas fewer taxa were detected in study XI due to the lower sequencing depth (Fig. S1B). *Clostridium* XIVa and *F. prausnitzii* of the Ac pathway were the only taxa observed in >90% of samples. Transcripts associated with *Oscillibacter* (Ac) were detected in 72% of samples (data not shown).

**FIG 2:**
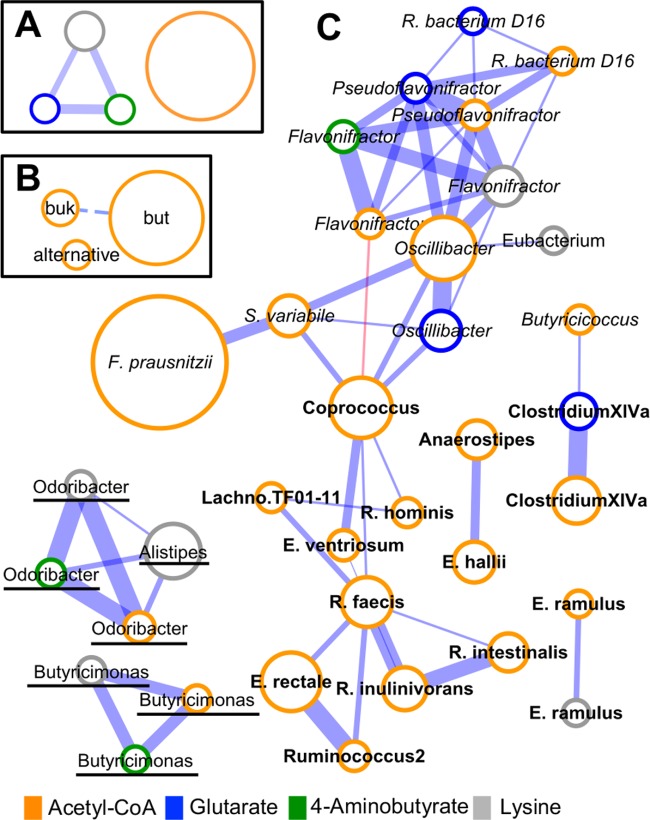
Correlation between abundances of butyrate-forming pathways (A), of terminal enzymes linked to the acetyl-CoA pathway (B), and of all individual taxa harboring a pathway (C). Analyses were performed on data derived from eight data sets (studies I to VI, VIII, and IX), where line width represents correlation strength, defined as the number of data sets displaying a correlation (*P* and *Q* < 0.05 and Spearman’s ρ > 0.4; a minimum of three correlations was required for connecting individual nodes; the dashed line in panel B represents correlations in two data sets). Node sizes reflect mean abundances (*n* = 813). Members of the *Lachnospiraceae* are indicated in bold, *Ruminococcaceae* are indicated in italics, and *Bacteroidetes* are underlined. Only taxa that were detected in >50% of samples were considered for analysis. but, butyryl-CoA:acetate CoA transferase; buk, butyrate kinase.

Cooccurrence analysis based on eight data sets (studies I to VI, VIII, and IX) revealed that abundances among protein-fed pathways correlated well (four to six correlations), whereas the Ac pathway showed a unique behavior (Fig. 2A). Overall, no correlations between groups expressing distinct terminal Ac pathway enzymes were observed (Fig. 2B); *but* and *buk* correlated in two data sets (I and VI), which was below the set threshold of ≥3 studies to record correlations. Nodes of individual taxa clustered according to their taxonomic affiliations, where members of the *Lachnospiraceae* and *Ruminococcaceae* grouped distinctly in the main network module. A few *Lachnospiraceae* and *Bacteroidetes* members formed small, separate groups (Fig. 2C).

Complete-linkage clustering of all samples (*n* = 826) resulted in low stratification for Ac pathway communities, and most samples displayed diverse compositions dominated by *F. prausnitzii*, yet a few specific community types enriched in certain taxa such as *Butyrivibrio crossotus*, *E. rectale*, *Oscillibacter*, and *R. faecis* were detected (Fig. S3A); for protein-fed pathways, clearly distinct community types were observed (Fig. S3B to D). Samples from individuals originating from distinct continents were not associated with any particular types of pathway communities.

10.1128/mSystems.00130-17.3 FIG S3  Stratification of pathway communities derived from healthy individuals of studies I to IX (*n* = 826). Samples are arranged based on results from complete-linkage clustering of individual pathway communities (acetyl-CoA [A], glutarate [B], 4-aminobutyrate [C], and lysine [D]). Relative abundances of major taxa and their taxonomic affiliations (bold, *Lachnospiraceae*; italic, *Ruminococcaceae*; underlined, *Bacteroidetes*) are given. Results from clustering are shown at the top of each panel, whereas origins of samples (continent) are indicated below. Download FIG S3, JPG file, 1.2 MB. Copyright © 2017 Vital et al. 2017 Vital et al. This content is distributed under the terms of the Creative Commons Attribution 4.0 International license.

### Succession of the butyrate-producing community after birth.

**FIG 3:**
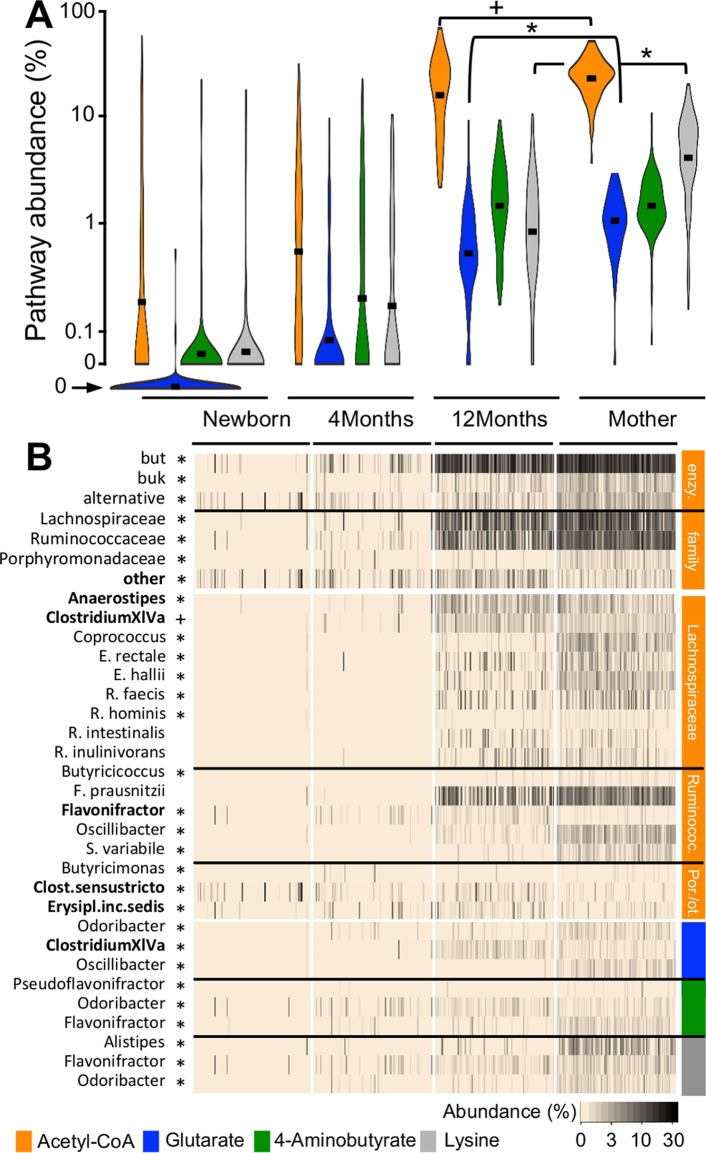
Succession of the butyrate-producing community after birth. Samples from mothers (*n* = 100) and their infants (1 week [*n* = 98], 4 months [*n* = 100], and 12 months [*n* = 100] after birth) were analyzed. (A) Abundances of bacteria exhibiting respective pathways as percentages of total bacteria (results for the glutarate pathway in newborns were manually shifted down to fit the plot layout). (B) Abundances of acetyl-CoA pathway groups, i.e., “enzyme” (cumulative abundance of all taxa exhibiting distinct terminal enzymes; enzy.) and “family” (cumulative abundance of all taxa of respective taxonomic families), as well as abundances of major individual taxa. Pathway affiliations of taxa are indicated by the color bars; members of the acetyl-CoA pathway are arranged on the family level. Significant differences (*P* < 0.05; *) and trends (*P* < 0.1; +) between mothers and their 12-month-old infants based on FDR-corrected pairwise Student *t* tests (pathway abundances) and Wilcoxon signed-rank tests (B) are illustrated; taxa enriched in 12-month-old infants are highlighted in bold. Black bars in violin plots represent mean values. Ruminococ., *Ruminococcaceae*; Por./ot., *Porphyromonadaceae*/other families.

In order to investigate the establishment of butyrate producers in newborns, metagenomes of samples from study VI (8) were analyzed involving 100 mothers and their infants sampled at 1 week, 4 months, and 1 year after birth (Fig. 3). A clear successional pattern of butyrate-producing communities in the first year of life was observed. Ac pathway abundances were low in newborns (1 week after birth) and 4-month-old individuals, where only seven and 14 subjects, respectively, harbored communities with >5% of bacteria exhibiting the pathway. Primarily, three genera, namely, *Clostridium sensu stricto*, *Erysipelotrichaceae incertae sedis*, and *Flavonifractor*, contributed to this pathway in those individuals (Fig. 3). In 4-month-old infants, these taxa were enriched in caesarean section-born individuals (compared with vaginally delivered children) and subjects who were formula fed (compared with breastfed infants) (Fig. S4A). Due to low abundances of butyrate producers in newborns, no analyses were performed in that group. In the majority of 1-year-old infants (86%), more than 5% of gut bacteria harbored the Ac pathway, a pattern similar to that of mothers, where 99% of samples exhibited communities with >5% of bacteria harboring this pathway, though the mean abundance still tended to be lower in infants at this age (Fig. 3). Protein-fed pathways showed a similar successional pattern as the Ac pathway, with low abundances after birth and substantial increases within the first 12 months of life; however, mean abundances of the Gl and Ly pathways were still lower in 1-year-old infants than in mothers (Fig. 3). Our results are supported by data from other studies that reported low concentrations of butyrate in feces from newborns while adult-like levels were observed in 9- to 12-month-old children (17).

10.1128/mSystems.00130-17.4 FIG S4  Succession of the butyrate-producing community after birth (study VI). Taxa that differed significantly in abundance with diet or delivery mode in children at the age of 4 months (FDR-corrected *P* < 0.05 from Kruskal-Wallis tests) are shown in panel A; at the top, mean abundances (as percentage of total bacteria) of three taxa—*Clostridium sensu stricto*, *Erysipelotrichaceae incertae sedis*, and *Flavonifractor*—harboring the acetyl-CoA pathway are displayed, whereas abundances of *Flavonifractor* additionally containing the 4-aminobutyrate (4A) and lysine (L) pathways are indicated below. No differences in taxon abundances with diet were observed in 12-month-old infants. Nonmetric multidimensional scaling (NMDS) analysis of communities from mothers and their infants (4 months and 12 months after birth) is displayed in panel B. Only samples exhibiting at least three taxa harboring butyrate-producing pathways were considered for analyses. Samples derived from infants receiving breastfeeding (at the age of 4 months) and any breastfeeding (at the age of 12 months) are highlighted in green. In panel C, Bray-Curtis dissimilarities of butyrate-producing communities between infants (at the age of 12 months) and their mothers as well as between all mothers are illustrated. Due to the low abundance of butyrate producers in newborns, no analyses were performed in that group. In NMDS plots, tips of lines illustrate individual samples, whereas ellipses represent standard deviations of points. Download FIG S4, JPG file, 0.6 MB. Copyright © 2017 Vital et al. 2017 Vital et al. This content is distributed under the terms of the Creative Commons Attribution 4.0 International license.

Although the butyrate-producing potential in 1-year-olds was similar to that of their mothers, the community structure was still distinct (Fig. S4B). *Anaerostipes*, *Clostridium sensu stricto*, *Erysipelotrichaceae incertae sedis*, and *Flavonifractor* were more abundant in children than in mothers, whereas most other butyrate-producing taxa were not fully established yet (Fig. 3). Accordingly, Bray-Curtis (BC) dissimilarities between mated pairs (12-month-olds/mothers) were higher (0.69 ± 0.14) than between-mother dissimilarities (0.49 ± 0.13) (Fig. S4C).

### Role in health and disease.

**FIG 4:**
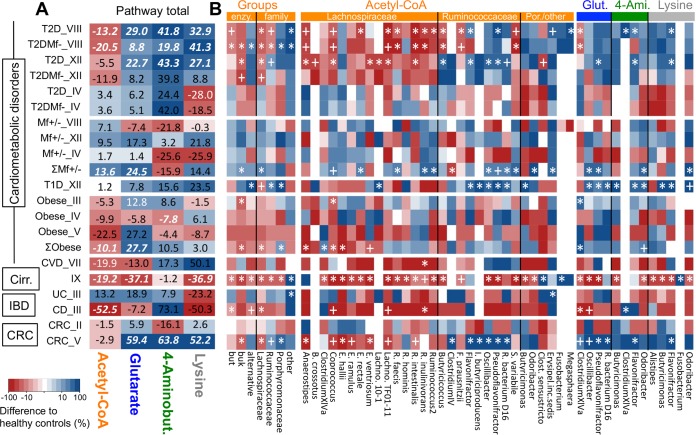
Alterations of the butyrate-producing potential in disease. (A and B) Abundances (defined as amounts of bacteria exhibiting respective pathways as percentages of total bacteria) of total pathways (A) as well as acetyl-CoA pathway groups, i.e., “enzyme” (cumulative abundance of all taxa exhibiting distinct terminal enzymes; enzy.) and “family” (cumulative abundance of all taxa of respective taxonomic families), and of individual taxa of all pathways (B) in diseased individuals relative to healthy controls (as percent; red, decrease; blue, increase). Eight data sets encompassing type 2 diabetes (T2D; studies IV, *n* = 43/53; VIII, *n* = 185/182; and XII, *n* = 293/75), obesity (III, *n* = 123/169; IV, *n* = 36/7; V, *n* = 57/7), type 1 diabetes (T1D; XII, *n* = 293/31), cardiovascular disease (CVD; VII, *n* = 13/12), liver cirrhosis (IX, *n* = 114/123), inflammatory bowel disease (IBD—ulcerative colitis [UC] and Crohn’s disease [CD]; III, *n* = 14/21/4, respectively), and colorectal cancer (CRC; II, *n* = 52/52; V, *n* = 66/91) were analyzed (*n* = *x*/*y* refers to sample sizes of healthy controls/patients, respectively). Values for total pathway abundance differences (A) that are highlighted in white, bold, and italic font represent significant changes to controls (*P* < 0.05; FDR corrected) from linear regression analysis, whereas simple white fonts show results that tended to be different (*P* < 0.1; FDR corrected). In panel B, significant differences (*, *P* < 0.05; +, *P* < 0.1) based on FDR-corrected Mann-Whitney U tests of acetyl-CoA pathway groups and of individual taxa are indicated (for the IBD data set, the bootstrapped version of the test was used due to small sample sizes). Pathway affiliations of taxa are indicated by the color bars; members of the acetyl-CoA pathway are arranged on the family level. Mf+/− refers to metformin-treated or untreated (+/−) samples (IV, *n* = 20/33; VIII, *n* = 15/56; XII, *n* = 58/17). The symbol Σ represents results of meta-analyses for metformin treatment (ΣMf+/−; IV, VIII, and XII) and obesity (ΣObese; III, IV, and V). Table 1 has the key to Roman numerals referring to individual data sets. Por./other, *Porphyromonadaceae*/other families; Glut., glutarate; 4-Ami., 4-aminobutyrate.

In order to obtain insights into the role of butyrate producers in health and disease, eight metagenomic data sets encompassing type 2 diabetes (T2D; studies IV [12], VIII [3], and XII [18]), obesity (study III [4]), cardiovascular disease (CVD; study VII [5]), liver cirrhosis (study IX [14]), inflammatory bowel disease (IBD; ulcerative colitis [UC] and Crohn’s disease [CD]) (study III [19]), and colorectal cancer (CRC; studies II [11] and V [13]) were analyzed (Fig. 4). For each data set, results are expressed as differences between diseased individuals and healthy controls (unless explicitly mentioned otherwise).

Mean abundances of the main butyrate-forming Ac pathway were reduced in T2D samples (compared with healthy controls) of studies VIII (−13.2% ± 4.8% [standard error {SE}]) and XII (−5.5% ± 6.8% [SE; *P* = 0.25]), in particular due to a decline of several *Lachnospiraceae*; in study VIII, a few abundant *Ruminococcaceae* (*F. prausnitzii* and *S. variabile*) decreased as well. The reduction was governed by taxa containing *but* and *buk*, whereas bacteria exhibiting “alternative” terminal enzymes were more abundant in T2D samples. Certain taxa of that pathway, such as *Oscillibacter* and *Pseudoflavonifractor*, together with (total) protein-fed pathways showed increased levels in T2D patients. Accordingly, the butyrate-producing community structure of T2D subjects was distinct from those of controls in both studies (Fig. S5A and B). Data set IV displayed a unique pattern where the butyrate-producing potential was not altered in disease, except for the Ly pathway that trended lower in T2D samples (*P* = 0.06). Pathway abundances in patients characterized by impaired glucose tolerance (study IV) were not different from those in healthy controls (data not shown). Treatment with metformin, a T2D therapeutic agent, resulted in elevated levels of the Ac pathway (compared with untreated T2D patients) in all data sets, with various key taxa such as *Coprococcus*, *R. inulinivorans*, and *S. variabile* consistently increasing. Significant changes (*P* < 0.05) were, however, observed only in a meta-analysis (Σ_IV,VIII,XII_) as sample sizes of individual studies were low. Community structures during treatment were altered as well (Fig. S5D). Accordingly, analyses comparing only untreated (metformin-negative) samples with healthy controls showed even higher reduction of butyrate producers in patient groups (in relation to results including all T2D samples), where mean abundances of the Ac pathway were reduced by 20.5% ± 7.0% (SE) and 11.9% ± 9.9% (SE) in studies VIII and XII (*P* = 0.22), respectively (Fig. 4). Type 1 diabetes (T1D) samples from study XII were analyzed to discriminate signatures of the gut microbiota specific to T2D from general influences of a glycemic phenotype (18). T1D had no effect on Ac pathway abundances, even though the community structure of bacteria harboring that pathway was profoundly changed, with several *Ruminococcaceae* increasing in abundance (Fig. 4). Communities derived from T1D individuals formed a unique cluster distinct from T2D and healthy control samples in nonmetric multidimensional scaling (NMDS) analyses (Fig. S5B).

10.1128/mSystems.00130-17.5 FIG S5  Nonmetric multidimensional scaling analyses of butyrate-producing communities in diseased individuals and healthy controls. Panels A to C display butyrate-producing communities derived from type 2 diabetes patients and healthy controls of studies VIII, XII, and IV, respectively. Samples from type 1 diabetes patients (XII) were included in panel B. The influence of metformin treatment on community structures (studies IV, VIII, and XII) is presented in panel D. Panel E illustrates communities derived from obese individuals from studies III, IV, and V compared with lean controls. Communities of atherosclerotic (study VII) and cirrhotic (study IX) individuals as well as from ulcerative colitis and Crohn’s disease patients (study III) compared with healthy controls are shown in panels F to H. Panel I displays butyrate-producing communities derived from colorectal cancer patients (II and V) and healthy controls. For the key to Roman numerals referring to individual studies, see Table 1. Patient communities that significantly differed from those derived from healthy controls (*P* < 0.05; *) or tending to be different (*P* < 0.1; +) based on permutational ANOVA are indicated. Tips of lines illustrate individual samples, whereas ellipses represent standard deviations of points. Download FIG S5, JPG file, 1.8 MB. Copyright © 2017 Vital et al. 2017 Vital et al. This content is distributed under the terms of the Creative Commons Attribution 4.0 International license.

Subjects suffering from T2D comorbidities, namely, obesity and cardiovascular disease (CVD), displayed reduced Ac pathway abundances (obesity, −10.1% ± 3.1% [SE] in meta-analysis [Σ_III,IV,V_], and CVD, −19.9% ± 12.2% [SE], *P* = 0.08) compared with healthy controls, mainly due to a decrease of certain *Lachnospiraceae* (Fig. 4). In obese individuals, alterations of the community structure were observed (Fig. S5E). The sample size of the CVD data set was small (*n* = 25), and despite large reductions observed for many taxa in the patient group, accompanied by significant alterations of the community structure (Fig. S5F), they were not found to differ statistically significantly.

The butyrate-producing potential was greatly reduced during liver cirrhosis (study IX), comprising three pathways (Ac, Gl, Ly) and a multitude of taxa, except for *Megasphaera* and *Fusobacterium*, which increased in samples from cirrhotic individuals compared with healthy controls (Fig. 4). Accordingly, the community structure in patients was profoundly altered (Fig. S5G).

Samples from UC and CD patients displayed distinct results where the mean abundance of the Ac pathway was reduced only in the latter patients compared with healthy controls (−52.5% ± 13.1% [SE], Fig. 4). Accordingly, the community structure was altered in those individuals (Fig. S5H). Sample sizes were small (UC, *n* = 21; CD, *n* = 4; healthy controls, *n* = 14), limiting the extraction of statistically robust signals.

In both CRC data sets, mean Ac pathway abundances in patients were unchanged from healthy controls. Only in study V was the community structure of CRC samples altered (Fig. S5I), where several *Lachnospiraceae* displayed lower levels in patient samples, which was balanced by an increase of *Ruminococcaceae* and bacteria affiliated with other families (Fig. 4). Protein-fed pathways were elevated in CRC patients from study V. Samples from subjects exhibiting adenomas showed similar pathway abundances as healthy controls, and no significant differences (from healthy controls) were found, except for the Gl pathway, which was increased by 33.3% ± 17.3% (SE) in individuals who had large adenomas (data not shown). For data set II, mean abundances of individual taxa in CRC samples did not differ from healthy controls and the community structure was not altered.

### Response of butyrate producers to disturbance events.

To investigate the behavior of butyrate-producing communities during disturbance, four data sets were analyzed including (i) antibiotic treatment (cefprozil for 7 days [study XIII {20}]) and two dietary intervention studies that monitored influences of (ii) plant-based versus animal product-based diets (3/4 days, study XI [16]) in healthy individuals and of (iii) a high-fiber, low-protein diet in obese children (up to 90 days, study XIV [21]) as well as one study that followed (iv) fecal transplantations from lean donors into obese, insulin-insensitive individuals (study XV [22]). Data sets XIII to XV comprised metagenomes, whereas study XI was based on metatranscriptomic data.

**FIG 5:**
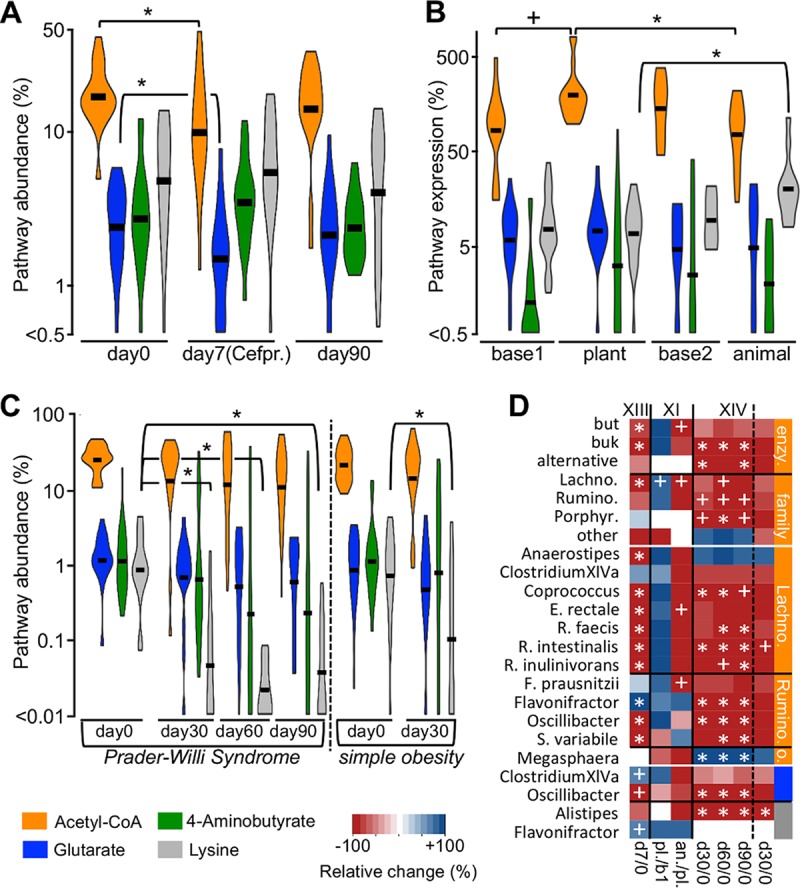
The butyrate-producing community during disturbance. (A) Abundances of bacteria exhibiting respective pathways as percentages of total bacteria in metagenomic data (pathway abundance) before (day 0) and after 7 days of antibiotic treatment [day 7 (Cefpr.); *n* = 18, study XIII] as well as 83 days after treatment (day 90). (B) Expression of butyrate-producing pathways based on metatranscriptomic data (relative to the mean expression of three housekeeping genes) during a dietary intervention study (XI) that subjected 10 individuals to a sequential plant (plant)- and animal-product (animal)-based diet is shown. Results of samples from individual subject-specific baseline diets (base 1 [before plant-based diet] and base 2 [before animal-product-based diet]) are included. (C) Pathway abundances (study XIV) derived from metagenomic data before (day 0) and during high-fiber, low-protein dietary interventions in Chinese children suffering from genetic obesity (Prader-Willi syndrome, *n* = 17) and diet-related “simple” obesity (*n* = 21) who were sampled before interventions (day 0) and after 30 days (day 30). Prader-Willi syndrome patients were additionally sampled at days 60 (day 60) and 90 (day 90). (D) Heat map showing abundance/expression changes of acetyl-CoA pathway groups, i.e., “enzyme” (cumulative abundance of all taxa exhibiting distinct terminal enzymes; enzy.) and “family” (cumulative abundance of all taxa of respective taxonomic families), and of major individual taxa during interventions. Pathway affiliations of taxa are indicated by the color bars; members of the acetyl-CoA pathway are arranged on the family level. In panel D, only abundance changes of taxa at day 7 (cefprozil treatment) compared with day 0 (d7/0) are shown for study XIII, whereas changes in gene expression associated with respective taxa between the plant-based diet and (i) either the first baseline diet (pl./b1) or (ii) the animal-product-based diet (an./pl.) are displayed for study XI. For study XIV, results from all time points (compared with day 0) are displayed. Significant differences (*P* < 0.05; *) and trends (*P* < 0.1; +) based on FDR-corrected pairwise Student *t* tests (A to C) and Wilcoxon signed-rank tests (D) are illustrated. Individual violin plots were manually shifted vertically to fit the plot size; black bars represent means. Lachno., *Lachnospiraceae*; Rumino., *Ruminococcaceae*; o., other families.

Antibiotic treatment profoundly influenced the butyrate-producing potential, reducing the Ac and Gl pathway levels in 13 and 15 (*n* = 18) individuals, respectively. Overall, mean abundances of those pathways were reduced by 35.6% ± 33.1% and 36.8% ± 33.9%, respectively, involving all terminal enzyme groups and most key taxa, except for *Flavonifractor*, which increased with treatment (Fig. 5A and D). Consequently, the community structure was altered and BC dissimilarities between communities before and after treatment were higher (0.38 ± 0.16) than BC dissimilarities derived from untreated controls over the same time period (0.18 ± 0.05; no treatment for 7 days) (Fig. S6A). Butyrate producers showed strong resilience, where by 83 days after the intervention (day 90) all pathways gained normal levels, with all taxa reaching abundances similar to those observed at baseline (Fig. 5A and S6A). However, BC dissimilarities between communities at day 0 and at day 90 (83 days after treatment) were higher (0.30 ± 0.15) than those of untreated controls (0.20 ± 0.06; no treatment for 90 days [Fig. S6A]).

10.1128/mSystems.00130-17.6 FIG S6  Analysis of the butyrate-producing communities during antibiotic treatment and during dietary interventions. Nonmetric multidimensional scaling (NMDS) analyses and heat maps showing abundances (as percentage of total bacteria)/expression (relative to three housekeeping genes [hkg]) of acetyl-CoA pathway groups, i.e., “enzyme” (cumulative abundance of all taxa exhibiting distinct terminal enzymes; enzy.) and “family” (cumulative abundance of all taxa of respective taxonomic families), and main individual taxa of all pathways are displayed. Panel A shows communities before (day 0) and after treatment [day 7 (cefprozil)] with the antibiotic cefprozil for 7 days (*n* = 18, study XIII) as well as results after 83 days of treatment (day 90). The expression of butyrate-producing pathways based on metatranscriptomic analyses during dietary intervention where 10 individuals were subjected to a sequential plant (plant)- and animal-product (animal)-based diet is shown in panel B. Samples from individual subject-specific baseline diets (base 1 [before plant-based diet] and base 2 [before animal-product-based diet]) were included (study XI; not all individuals were sampled at every time point). Bray-Curtis dissimilarities of pairwise comparisons between individual groups within subjects are displayed. Dissimilarities of untreated controls (7 days [control_d0/d7] and 90 days [control_d0/d90]) from study XIII and dissimilarities between individuals within different groups (for study XI) are shown as well. Significant differences (*P* < 0.05; *) and trends (*P* < 0.1; +) based on FDR-corrected pairwise Wilcoxon signed-rank tests for before-after treatment (study XIII) and plant versus base 1 (b1/pl.) or animal diet (an./pl.) (study XI) comparisons are illustrated. Pathway affiliations of taxa are indicated by the color bars; members of the acetyl-CoA pathway are arranged on the family level. *Lachnospira*., *Lachnospiraceae*; Ruminococ., *Ruminococcaceae*; o., *Porphyromonadaceae*/other families. In NMDS plots, communities derived from individual subjects are connected and ellipses represent standard deviations of points. Download FIG S6, JPG file, 1.3 MB. Copyright © 2017 Vital et al. 2017 Vital et al. This content is distributed under the terms of the Creative Commons Attribution 4.0 International license.

Expression of butyrate-producing pathways was sensitive to dietary interventions, as the Ac pathway was upregulated during plant-based diets compared with animal product-based nutrition (Fig. 5B). Expression during the former diet also tended to increase (*P* = 0.07) over baseline samples. Accordingly, measured butyrate concentrations were reduced in samples derived from diets rich in animal products compared with the plant-based diet (16), despite increased expression of the Ly pathway. Community structures were altered during interventions, and the animal product-based nutrition resulted in higher dispersed communities (BC, 0.70 ± 0.16) compared with samples from plant-based (BC, 0.49 ± 0.19) and baseline (BC, 0.47 ± 0.12) diets (Fig. S6B). Gene expression of some taxa tended to decrease during the animal product-based diet compared with plant-based nutrition (*P* < 0.1) (Fig. 5D). However, the number of pairwise comparisons was low (*n*_max_ = 8), and despite the reduced expression of genes of major *but*-containing taxa, namely, *Coprococcus*, *E. rectale*, *F. prausnitzii*, and *R. faecis*, during the former diet in all individuals (expression levels of genes associated with *R. intestinalis* and *R. inulinivorans* were higher in only one subject), changes did not reach statistical significance (*P* > 0.05).

Longer-term interventions based on high-fiber, low-protein diets in obese children suffering from diet-related (“simple”) obesity (SO; 30 days, *n* = 21) and Prader-Willi syndrome (PWS; 90 days, *n* = 17) did not significantly affect mean abundances of the Ac pathway in either group (Fig. 5C). However, a decrease of many major taxa was detected after 30 days, in particular in PWS samples, and those taxa maintained low abundances throughout the experiment (Fig. 5D). Conversely, *Anaerostipes* and *Megasphaera* increased in several individuals, reaching high abundances (>5% of the total community) in 3/2 (PWS/SO) and 5/7 (PWS/SO) individuals, respectively, sustaining similar overall abundances of the Ac pathway throughout the experiment (Fig. S7). Analytical measurements were in line with these results, where the mean concentration of butyrate did not significantly change with treatment (21). Mean abundances of the Ly pathway were highly reduced after 30 days in both patient groups and remained low throughout the experiment.

10.1128/mSystems.00130-17.7 FIG S7  Nonmetric multidimensional scaling (NMDS) analysis of butyrate-producing communities (A) during dietary interventions treating Chinese children suffering from genetic obesity (Prader-Willi syndrome [PWS]; *n* = 17) and diet-related “simple” obesity (SO; *n* = 21) with a high-fiber, low-protein diet (study XIV). Samples were taken before (day 0) and after 30 days, as well as after 60 and 90 days (only for PWS) during interventions. Bray-Curtis dissimilarities of pairwise comparisons between individual groups are displayed in panel B. The heat map (C) shows abundances (as percentages of total bacteria) of acetyl-CoA pathway groups, i.e., “enzyme” (cumulative abundance of all taxa exhibiting distinct terminal enzymes; enzy.) and “family” (cumulative abundance of all taxa of respective taxonomic families), and of main individual taxa of all pathways. Significant differences (*P* < 0.05) in taxon abundances compared with controls (before intervention, day 0) based on FDR-corrected pairwise Wilcoxon signed-rank tests are illustrated as colored dots in panel B, where no edge indicates a decrease during interventions and black edges illustrate significant increases. In SO patients, only the abundance of *Alistipes* changed significantly (blue diamond). Pathway affiliations of taxa are indicated by the color bars; members of the acetyl-CoA pathway are arranged on the family level. *Lachnospira*., *Lachnospiraceae*; Ruminococ., *Ruminococcaceae*; Por./oth., *Porphyromonadaceae*/other families. Ellipses in NMDS plots represent standard deviations of points. Download FIG S7, JPG file, 1.4 MB. Copyright © 2017 Vital et al. 2017 Vital et al. This content is distributed under the terms of the Creative Commons Attribution 4.0 International license.

Communities of obese, insulin-insensitive patients who received fecal transplants were highly dissimilar before the experiment and responded distinctly to the intervention (Fig. S8A). In two (P6 and P8) of the five patients analyzed, the community structure changed profoundly after transplantation and displayed closer similarities to the donor communities at 2 days postintervention. In P6, communities were approaching their original structure over the 84 days of the experiment (BC dissimilarity with the original community before transplantation decreased over time [Fig. S8B]), whereas those from P8 displayed compositions distinct from both the donor and the initial community over time. Communities of P12 and P15 were less affected by the transplantation and displayed diffuse responses over the duration of the experiment, whereas the community of P20 was moderately altered after the intervention and clustered between the donor and baseline communities (Fig. S8A).

10.1128/mSystems.00130-17.8 FIG S8  Nonmetric multidimensional scaling analyses of five fecal transplant patients (different shades of blue) and their donors (green) are illustrated (A; study XV). Samples were taken before transplantation (day 0; orange circles) and 2, 14, 42, and 84 days after intervention. Red dashed lines connect donor communities with communities of recipients 2 days after transplantation. Bray-Curtis (BC) dissimilarities between the butyrate-producing community of each sample and (1) the donor community (blue) as well as (2) the original community (day 0, red) are given in panel B. Download FIG S8, JPG file, 0.6 MB. Copyright © 2017 Vital et al. 2017 Vital et al. This content is distributed under the terms of the Creative Commons Attribution 4.0 International license.

### Diversity and functional stability.

**FIG 6:**
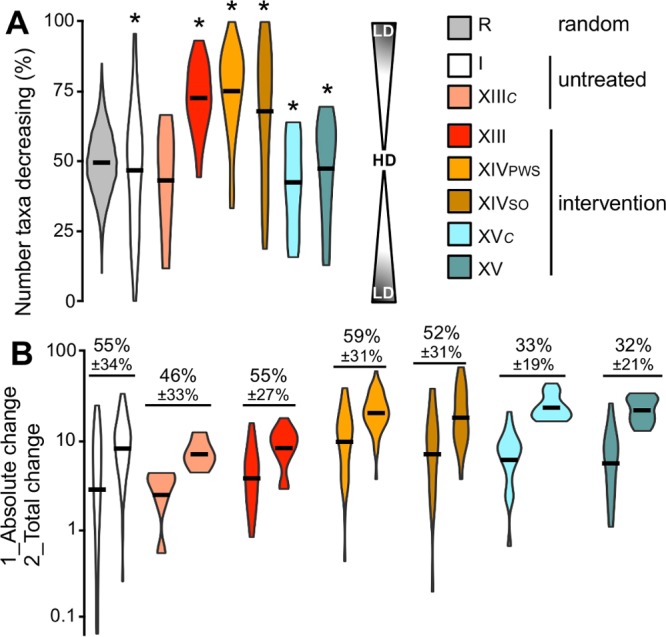
Influence of Ac pathway diversity on temporal stability (untreated individuals) and during interventions. Data derived from the Human Microbiome Project (study I), antibiotic-treated individuals (XIII) and their untreated controls (XIII_*C*_), and dietary interventions in Chinese children suffering from Prader-Willi syndrome (XIV_PWS_) and diet-related “simple” obesity (study XIV_SO_) as well as from autologous (XV_*C*_) and allogenic (XV) transplant patients were included in the analyses. Calculations were based on abundance differences of taxa between the initial time point and all subsequent time points in each subject (I, *n* = 53; XIII, *n* = 18; XIII_*C*_, *n* = 6; XIV_PWS_, *n* = 51; XIV_SO_, *n* = 20; XV_*C*_, *n* = 20; XV, *n* = 20). (A) Percentage of taxa that decreased in abundance over time within each individual. Results from random communities (R, gray violin plots), constructed by 20 random samplings (function *sample* in R; range, −100 to +100; *n* = 1,000), are included. A schematic representation from highest discordance (HD; half of the taxa decrease, whereas the other half increase) to lowest discordance/highest concordance (LD; all members change in the same direction) is indicated. Variances, i.e., deviations from 50%, that were significantly higher than those in random communities are indicated (*, *P* < 0.05; Student’s *t* tests). (B) “Absolute changes,” i.e., cumulative absolute abundance changes of individual taxa between two time points including the direction (decrease/increase), which represents the overall abundance change of the entire pathway (directions of final cumulative abundance changes were omitted; hence, all values were ≥0), with “total changes,” i.e., cumulative absolute abundance change of individual taxa disregarding the direction. In the panel, results of “absolute changes” are followed by results of “total changes” for each data set. The percentages of “absolute changes” from “total changes” (means ± standard deviations) are printed for all data sets. For explanations, see the text. Black bars in violin plots represent mean values.

The highly diverse butyrate-producing communities revealed, in particular those associated with the Ac pathway, led us to investigate in more detail the relationship between the individual behavior of functionally redundant taxa and the overall stability of this pathway. All metagenomic data sets that sampled subjects over time, namely, studies I (HMP [10]), XIII (antibiotic treatment [20]), XIV (dietary intervention [21]), and XV (fecal transplantation [22]), were included in the analyses. Abundance changes for each taxon within each subject were calculated from absolute abundance (percentage of total bacteria) differences between the initial time point and all subsequent time points. Taxa in subjects not exposed to any intervention (untreated individuals; HMP [study I] and control subjects of study XIII) showed dissimilar behavior where on average ~50% of taxa decreased in abundance over time (with the other half concurrently increasing [Fig. 6A]). However, variances were high (results deviated from a 50% decrease, which represents the highest dissimilarity) and more pronounced than random fluctuations (untreated controls of study XIII displayed 1-fold-higher mean variance than random communities; however, results were not significantly different due to the small sample size [*n* = 6]). Thus, while butyrate-producing taxa behaved highly disparately in untreated individuals, the results suggest that their responses to (unknown) temporal alterations in the gut environment were partly correlated (concurrent decreases/increases). Individuals subjected to specific interventions, namely, antibiotic treatment (study XIII) and dietary interventions (study XIV), displayed strongly directed behavior where the bulk of taxa (~70 to 75%) decreased (Fig. 6A). Community responses of autologous and allogenic transplant patients displayed similar patterns as untreated subjects.

We further compared the “absolute change,” i.e., the cumulative absolute abundance change of individual taxa between two time points including the direction (decrease/increase), which represents the overall abundance change of the entire pathway (the direction of the cumulative abundance change was omitted), with the “total change,” i.e., the cumulative absolute abundance change of individual taxa disregarding the direction (Fig. 6B). If all taxa are changing in the same direction (abundances of all taxa decrease or increase), results from the two calculations will be equal. Since absolute abundance changes were only a fraction (32% to 59%) of total abundance changes in all data sets, our data suggest that disparate behavior of butyrate producers supported functional stability.

## DISCUSSION

In this study, we provide a comprehensive overview of the butyrate-producing communities of the human colon. Overall, our results do largely agree with findings reported in original studies (see Table S1 in the supplemental material); however, pooled analyses comprising various data sets allowed detailed insight into the ecology of butyrate producers and specification of their role in health and disease.

Butyrate-producing communities displayed universal patterns irrespective of data set origin where the Ac pathway was dominant over protein-fed pathways. The bulk of samples contained diverse communities comprising various taxonomically distinct butyrate producers with 17 specific taxa that were present in the majority of individuals (>70%), encompassing ~85% of the total butyrate-producing potential; a few taxa were even detected in >90% of subjects, including *F. prausnitzii*, which dominated in all data sets, constituting roughly one-fourth of all butyrate producers exhibiting the Ac pathway. Metatranscriptomic data demonstrated that most of the detected key taxa were also expressing pathways, indicating that a multitude of distinct bacteria contribute to the butyrate pool in the human colon. Furthermore, we observed primarily positive abundance correlations between taxa (Fig. 2) and low stratification of Ac pathway communities (Fig. S2A), suggesting limited competition between major butyrate producers and only minor niche overlaps (at least at the genus/species level considered here). In conclusion, our data indicate that in healthy individuals a highly diverse butyrate-producing community occupies various niches of the gut ecosystem, collectively providing a high potential to synthesize that compound. It should be mentioned that only subjects from healthy control groups were included in analyses presented in Fig. 1 and 2 (a few controls knowingly suffering from disorders such as obesity were omitted as well); however, not all subjects can be regarded as being exclusively healthy due to scarce metadata and different exclusion criteria between studies. Furthermore, sampling procedures and nucleic acid extraction techniques differed between studies, which might have introduced some data set-specific biases.

The high taxon richness observed, especially for the Ac pathway, indicates great functional redundancy. However, the extent to which individual butyrate producers are indeed redundant depends on their biochemical characteristics. Most importantly, we consider redundancy of individual pathways as limited since the Ac pathway is a major fermentative route for bacteria transforming vast amounts of substrate, whereas substrate fluxes via protein-fed pathways are expected to be of minor importance (9). Thus, the Ac pathway dominates butyrate synthesis, and a reduction of this main pathway can barely be balanced by increasing abundance or expression of other pathways. Data obtained from dietary interventions (study XI) support this assumption, where decreased levels of the Ac pathway during protein-rich diets resulted in reduced butyrate concentrations (16) despite an observed increase of the protein-fed Ly pathway. Limited redundancy between pathways is specifically important when interpreting the results obtained from data sets focusing on cardiometabolic disorders, where detected increases in protein-fed pathways in diseased individuals are of minor importance and should not distract from the observed reduction of the main Ac pathway.

Within each pathway, we consider bacteria largely functionally redundant, and our data propose that distinct behavior of taxa supports functional stability during ordinary life disturbances and during specific interventions (Fig. 6). While most butyrate producers declined during interventions, the distinct behavior of a few taxa helped attenuate the decrease of the overall pathway abundance and sometimes even sustained the butyrate production potential. This is exemplified by *Megasphaera*/*Anaerostipes*, which, in contrast to most other taxa, increased during dietary intervention (study XIV), maintaining the Ac pathway at similar levels throughout the experiment in several individuals (Fig. 5C and S7C). Furthermore, taxa were highly resilient after treatment (study XIII), although the intervention period was moderate in length (7 days), and investigations on restoration of butyrate-producing communities after longer-term (“press”) disturbances (23), such as prolonged hospital stays or extended dietary interventions, are needed. Taxa in individuals not subjected to any specific interventions (the HMP [study I] and control subjects of study XIII) behaved disparately (Fig. 6), possibly reflecting adaptive features of the functional community to cope with temporal environmental changes. However, pathway abundances were not completely stable, and butyrate producers partly showed correlated responses that exceeded random fluctuations, suggesting that the butyrate production potential is sensitive to variations from everyday life. Overall, we propose stabilizing effects of redundant, diverse communities on the functional potential during disturbance, as considered earlier (24). To what extent diversity correlates with functional stability and the role of specific community compositions still needs to be addressed in detail.

Our study is primarily based on metagenomic data that represent the butyrate-producing potential, which limits predictions on actual butyrate synthesis. However, conversion of acetyl-CoA to butyrate is a metabolic cornerstone of most bacteria exhibiting that pathway, as it serves as a main fermentative route during growth. The presence of the Ac pathway does, hence, indicate activity, which is supported by metatranscriptomic data in this study. In line with our findings, a recent metaproteomic study revealed *F. prausnitzii* as the main taxon expressing that pathway in all individuals analyzed (*n* = 15) (25). Furthermore, tight correlations between abundances of Ac pathway-containing bacteria and actual measured concentrations of butyrate were reported in a study culturing whole-gut communities (26). Protein-fed pathways, on the other hand, are not essential for growth of most taxa in the gut, and their presence is less tightly coupled to activity, which is reflected in results obtained from dietary interventions based on a low-protein diet (study XIV). Only the Ly pathway significantly declined upon depletion of proteins, whereas the Gl and 4A pathways were less affected and sustained similar levels throughout the experiment, indicating alternative carbon/energy sources for bacteria exhibiting the latter pathways. In fact, all major taxa exhibiting those pathways also harbor the carbohydrate-based Ac pathway, providing metabolic flexibility for butyrate synthesis. Eventually, multiomics analyses together with measurements of growth substrates and concentrations of fermentation end products will allow uncovering the dynamics of butyrate production *in vivo* and revealing the contribution of each taxon in detail.

This study supports the general view that sufficient butyrate production by the gut microbiota is crucial for maintaining host health, where imbalances in its supply can promote disease (27). Reductions of the Ac pathway along with a decline of several key butyrate-producing taxa were detected in patients, in particular those suffering from cardiometabolic disorders. For type 2 diabetes, the insights gained reach beyond associative observations, as gut communities of T1D patients were largely unaffected, demonstrating that the phenotype alone, i.e., high glycemia, was not responsible for the observed reduction of the Ac pathway in T2D individuals. In line with the original study (18), metformin treatment resulted in an increase of several key taxa harboring the Ac pathway, suggesting that besides its primary function of inhibiting glucose production in the liver, stimulation of gut communities to produce butyrate contributes to the therapeutic effects. Individuals suffering from comorbidities, namely, obesity and atherosclerosis, showed a reduced potential for butyrate synthesis as well (Fig. 3). Thus, in accordance with results from animal studies (cf. references 28 and 29), there is strong evidence that a decline in butyrate concentration plays a role in the etiology of cardiometabolic disorders. It is proposed that chronically reduced levels of butyrate contribute to gut barrier disruption and metabolic endotoxemia, promoting low-grade inflammation that leads to disease (30, 31). The role of butyrate in the etiology of other diseases analyzed here is less clear. For instance, despite a pronounced reduction of butyrate producers in patients suffering from liver cirrhosis, it remains elusive whether this reduction was indeed promoting the phenotype or whether alterations in host metabolism were subsequently affecting the composition of the gut microbiota. Similarly, the reduced butyrate-producing potential detected in Crohn’s disease (CD) patients did not reach beyond associative insights yet. A pioneer metagenomics/metaproteomics study (*n* = 12) that distinguished between CD in the ileum and the colon also reported lower abundances of genes for butyrate formation along with their reduced expression in both patient groups compared with healthy controls (32). Reduction of the key enzyme butyrate transferase was more pronounced in subjects suffering from ileal CD, and data from larger cohorts will allow extraction of detailed differences between the two groups in future. We did not find evidence that (a reduced level of) butyrate plays a role in the development of colorectal cancer, which is in accordance with the original studies (11, 13).

Only a few taxa were reduced across various diseases (e.g., *E. rectale*/*Coprococcus*), whereas others displayed diverse behavior. For instance, several *Roseburia* species decreased in T2D samples; however, their abundances were hardly affected in obese individuals. Abundances of other butyrate producers were not altered in any disease or even displayed increased levels (e.g., *Flavonifractor*). This highlights the complex nature of functional dysbioses that are based on taxonomically diverse, functionally redundant communities and demands precision treatment to combat disease. Administration of abundant, barely reduced taxa is most unlikely to be a promising approach, and interventions specifically boosting various niches of highly reduced key taxa appropriately filling the entire spectrum of this functional group in each individual will be superior in increasing butyrate production effectively. In any case, establishing a diverse, functionally redundant community that has the potential to adjust to specific (temporal) conditions should be considered for the development of sustainable treatment strategies.

## MATERIALS AND METHODS

### Updating the database.

We updated a previously published butyrate synthesis gene database, applying the original workflow encompassing a multilevel screening approach (7) with some modifications. New hidden Markov models (HMM) on full-length proteins considering the entire taxonomic diversity of butyrate producers revealed earlier (7) were constructed (*hmmbuild* default mode, HMMER 3.1b1; http://hmmer.org/) and used to screen (*hmmsearch*) 67,134 genomes provided by PATRIC (https://www.patricbrc.org; April 2016) for the presence of respective pathway genes (*n* = 27, Fig. 1A). Genomes derived from metagenomes were not considered. For each protein, sequences were sorted based on similarity scores to the model, and low-score cutoffs at the end of obvious score drops after the lowest-scoring reference protein were set. All sequences above those cutoffs were considered for follow-up analyses. Genomes were filtered for exhibiting entire pathways. Genes encoding the terminal enzymes butyryl-CoA:acetate CoA transferase (But), butyryl-CoA:4-hydroxybutyrate CoA transferase (4Hbt), and butyrate kinase (Buk) were separately analyzed as described previously (7). A pathway was recorded as being present if none or only one pathway gene was absent. For the Ac pathway, candidates had to exhibit a terminal enzyme (But/Buk) if lacking other genes of that pathway; the presence of thiolase (acetyl-CoA acetyltransferase, *thl*) was no selection criterion, as some butyrate producers have alternative routes for the formation of acetoacetyl-CoA (7, 33). Finally, paralogs localized in genomes without synteny with other pathway genes (defined as being separated by ≤10 genes based on locus tag) and displaying lower HMM scores than the respective syntenic genes were omitted. *etfAB* genes encoding electron transfer proteins were considered only if they showed synteny with *bcd*, encoding butyryl-CoA dehydrogenase, indicating that they encoded the bifurcating butyryl-CoA dehydrogenase complex (*bcd-etfAB*). Our database now contains 1,716 genomes encompassing 19,284 genes, and all sequences can be downloaded at http://193.175.244.101/Butyrate/.

HMMs for the housekeeping genes encoding 50S ribosomal protein L2 (*rplB*), recombinase A (*recA*), and CTP-synthase (*pyrG*) were retrieved from FunGene (34) and used to screen genomes. All proteins above an obvious similarity score-drop were considered. Sequences of those genes were unique, displaying no similarities with any other gene. Genes were detected in 97%, 96%, and 94% of genomes at an average copy number of 1.03, 1.04, and 1.03 for *rplB*, *recA*, and *pyrG*, respectively. Taxonomic affiliations were based on the RDP taxonomy, where 16S rRNA gene sequences of genomes were retrieved and subjected to classification using the RDP classifier (35) as described previously (36).

### Processing of sequence data and establishing a gene catalogue.

Raw reads of all samples analyzed here were downloaded from the European Nucleotide Archive (http://www.ebi.ac.uk/ena) and quality filtered for an average *Q* score of ≥20 and length of ≥70 (for a few samples [*n* = 21] of study III [19], the length filter was adjusted to the provided read length of 44) using Trimmomatic (37). Data from files that derived from the same sample were merged. For constructing the gene catalogue, gene sequences for all pathway genes and housekeeping genes of individual samples were obtained using RDP’s gene-targeted assembler in default mode (kmer size of 45 [for the samples {*n* = 21} of study III mentioned above, kmer size was reduced to 42], a minimum count of two, and a minimum contig size of 150 amino acids [aa]), where the filter size was adjusted to ensure a false discovery rate (FDR) below 1% as suggested by the developers (38). For the gene *kal* (3-aminobutyryl-CoA ammonia-lyase) of the Ly pathway, the minimum contig size (90 aa) and bit score (30) were reduced to consider its short length (median, 130 nucleotides). In order to maintain all analyses on the nucleotide level, the following steps were performed outside the Xander pipeline. Merged nucleotide sequences (*nucl_merged*) from all samples were dereplicated, filtered for a length of ≥70% median gene length of respective reference sequences from genomes, and subjected to chimera removal (UCHIME [v. 4.2.40] using HMM seed sequences as references [39]) and FrameBot analysis (v. 1.2, in default mode, with all dereplicated references obtained from genome screenings [40]). For subsequent complete-linkage clustering (default mode [41]), proteins were aligned to the HMMs and obtained alignments served as references to align the corresponding nucleotide sequences, which were then subjected to clustering (at 95% nucleotide identity). Reference sequences derived from genome screenings above were included at this step. Representative sequences from clusters that were lacking any genomic reference sequences and contained ≥3 counts were subjected to a BLAST search (blastn, v. 2.2.28+; the top hit was recorded) against reference genes obtained from genome screenings (see above), and all sequences that displayed a coverage of ≥80% to references were included in our gene catalogue. For *but* and *buk*, an additional filtering step was included where only sequences with ≥75% nucleotide similarity to the top BLAST hit were considered. Finally, sequences were annotated according to the BLAST results and merged with all dereplicated nucleotide reference sequences, providing the gene catalogue for subsequent analysis.

### Analysis of butyrate-producing community in individual data sets.

Quality- and size-filtered reads were mapped to our gene catalogue using Bowtie 2 (v. 2.2.3, option --*very-sensitive*) (42). Paralogs as well as sequences located above the similarity score threshold (see above) from genera not containing any butyrate producers (mainly derived from *Bacilli* [~70%] and *Enterobacteriaceae* [>20%]) were included at this step, and reads that mapped to those sequences were filtered out in order to avoid the possibility of false-positive counts derived from those genes. Mapped reads were retrieved by SAMtools (v. 0.1.19, SAMtools view -S -F 4 [43]).

All genes of a pathway (excluding terminal genes and *bcd-etfAB*, which is present in all pathways) were used to calculate pathway abundances except for *gcdB* (encoding the beta subunit of glutaconyl-CoA decarboxylase) of the Gl pathway (recruitment of many false positives) (7). Results were gene length corrected using the median length of respective reference sequences and are presented as abundances (mean of all pathway genes) relative to mean abundances of the three housekeeping genes. As reported previously (7), obtained gene counts were very similar among genes of the same pathway, demonstrating accurate quantification of pathway abundances. The 4A pathway was an exception, where *abfD* (encoding 4-hydroxybutyryl-CoA dehydratase/vinylacetyl-CoA delta-isomerase) recruited proportionally high numbers of reads, and the median of length-corrected read counts was used for calculating its abundance.

To analyze taxonomic compositions of individual pathways, sequences from each gene of our catalogue derived from the same genus were binned together. Manual inspections of major, abundant genera led us to resolve *Roseburia* and *Lachnospiraceae incertae sedis* at the species level, as sequences of individual species displayed high phylogenetic distances for all pathway genes (>10% nucleotide dissimilarity, except for *thl* of *R. intestinalis* and *R. inulinivorans*, which showed a distance of 9.2%). This was also the case for *B. crossotus* compared with other *Butyrivibrio* spp. For genera that encompassed only one species, such as *F. prausnitzii* and *S. variabile*, the species name is displayed. Genomes that could not be classified at the genus level were treated separately. A complete list of PATRIC identifiers (IDs) associated with individual taxonomic bins is presented in Table S2A in the supplemental material. RDP taxonomy follows Bergey’s Trust, where the genus *Clostridium* XIVa is distinct from the *Clostridium* XIVa cluster as defined by Collins et al. (44) and does not encompass major butyrate-producing taxa such as *Roseburia* and *Coprococcus* that form separate genera. Although members of major taxa presented in Fig. 1 displayed similarities in presence and organization of pathways, variations between strains of certain taxa were detected, in particular for protein-fed pathways (Table S2A), demonstrating limitations for functional predictions solely based on taxonomic data (Table S2B).

10.1128/mSystems.00130-17.10 TABLE S2  In sheet A, the presence and organization of pathways in all candidates are shown, whereas the presence of pathways in genomes of all members of individual genera (based on all genomes derived from PATRIC) that are important for this study is given in sheet B. Download TABLE S2, XLSX file, 0.6 MB. Copyright © 2017 Vital et al. 2017 Vital et al. This content is distributed under the terms of the Creative Commons Attribution 4.0 International license.

A taxon was considered present if all genes of a pathway were detected for that taxon. The absence of specific pathway genes in certain taxa was accounted for based on data from genomic references. Due to the low sequencing depth of metatranscriptomes of data set XI (16), one missing gene for each taxon was allowed in that data set. The presence of *gctA* (Gl pathway; in some cases taxon reads matched all pathway genes except *gctA*) as well as *atoA/D* (only very low counts were obtained as many taxa are devoid of those genes) and *kal* (its short length limited detection of low-abundance taxa) of the Ly pathway was not required. Taxon abundances (percentage of total community) were calculated based on gene-length-corrected median read counts of all pathway genes compared with mean read counts of housekeeping genes. As for overall pathway calculations, length-corrected gene counts were largely consistent between all genes of a pathway within a taxon. The proportions of members from the same family (*Lachnospiraceae*, *Porphyromonadaceae*, *Ruminococcaceae*, and “others”) exhibiting the Ac pathway as well as the proportion of bacteria exhibiting distinct terminal enzymes (encoded by *but*, *buk*, and others [termed “alternatives”]) were calculated as well. The abundance of taxa containing both *but* and *buk* was proportionally split according to obtained abundances of each terminal gene. Similarly to our previous study (7), high percentages of reads that were used to calculate overall pathway abundances were linked to a taxon, namely, 91.5% ± 2.8%, 91.3% ± 5.0%, and 85.2% ± 7.6% for the Ac, Gl, and Ly pathways, respectively. For the 4A pathway, values were lower (60.4% ± 14.1%, excluding *abfD*).

Bray-Curtis dissimilarities, nonmetric multidimensional scaling (*metaMDS*), and permutational analysis of variance (ANOVA) (*adonis*) were calculated in R (v. 3.1.2) using the package vegan (v. 2.3 to 4) and were based on square-root-transformed relative abundance data of the entire butyrate-producing community (all taxa from all pathways were considered). Heat maps were created in R using the package gplots (v. 2.17.0) on log-normalized data [log(*x* + 1)]. FDR-corrected Mann-Whitney U and Kruskal-Wallis tests were done in QIIME (v. 1.9.1 [45]) considering all taxa that were present in ≥25% of samples, as suggested by the developers. Other statistical analyses were performed in R (Spearman correlation [package *Hmisc*], linear regression [*lm*], paired Wilcoxon signed-rank test [*wilcox.test*, paired=TRUE], paired Student’s *t* test [*t.test*, paired=TRUE], FDR correction [*p.adjust*], *Q* values [package *fdrtool*], and testing for normal distribution [*shapiro.test*]). All numbers following the ± sign are standard deviations, except for results derived from comparisons with healthy controls (Fig. 4), where the standard error of the difference is given (indicated as SE). Paired tests were performed only for taxa allowing for ≥5 pairwise comparisons (excluding tied zeros). Violin plots were constructed in R using the package easyggplot2. The network was visualized in Cytoscape (v. 2.3.1; http://cytoscape.org; preferred layout with some modifications) considering correlations (*P* and *Q* < 0.05, Spearman’s ρ ≥ 0.4) that were detected in at least three data sets (*n* = 8).
