# Supplementary figures and images for: Colonic Butyrate-Producing Communities in Humans: an Overview Using Omics Data
Source: mSystems. 2017 Dec 5;2(6):e00130-17. doi: 10.1128/mSystems.00130-17 (PMC5715108; doi:10.1128/mSystems.00130-17)

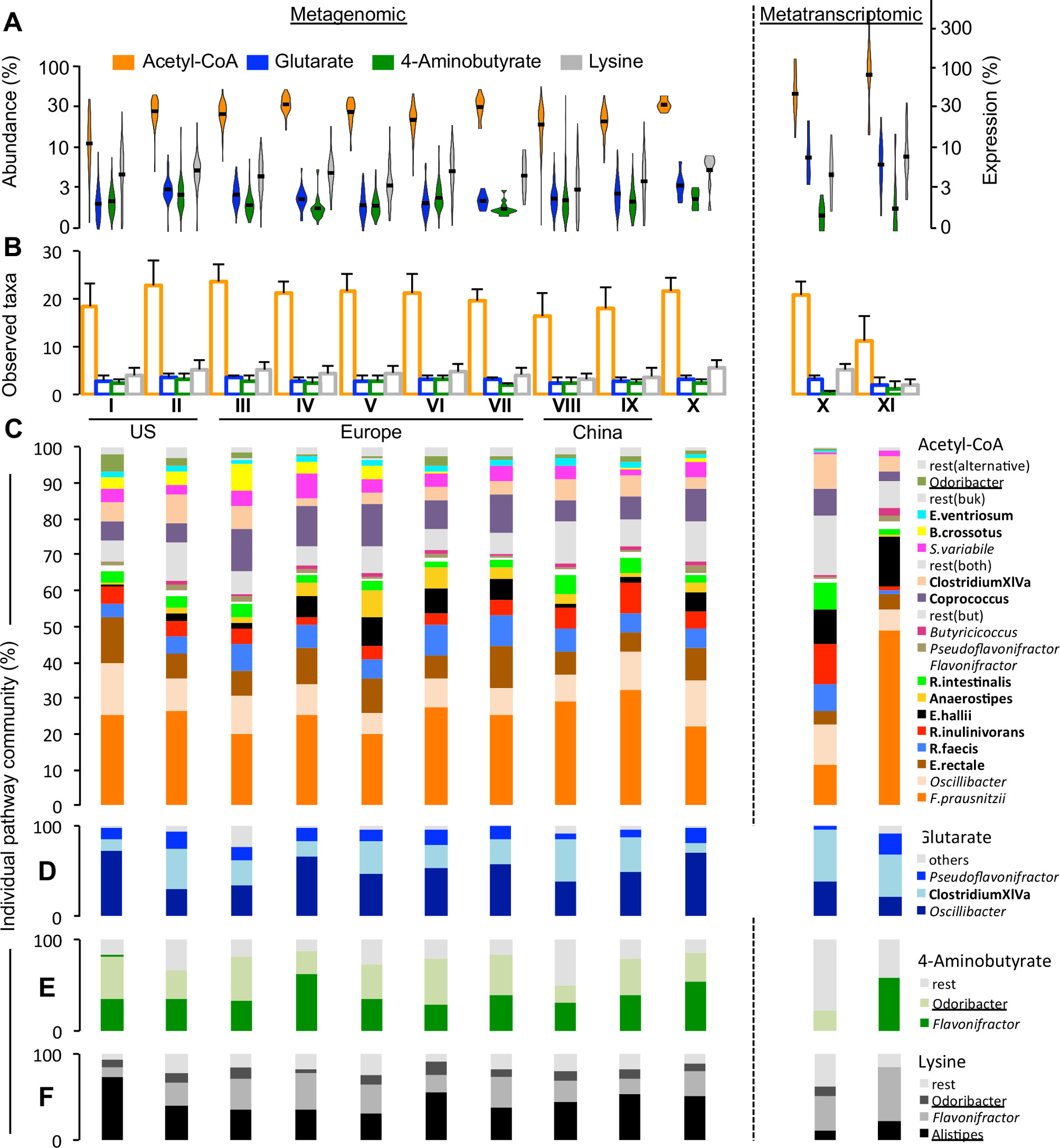

Supplement: FIG S1 [file sys006172157sf1.jpg]

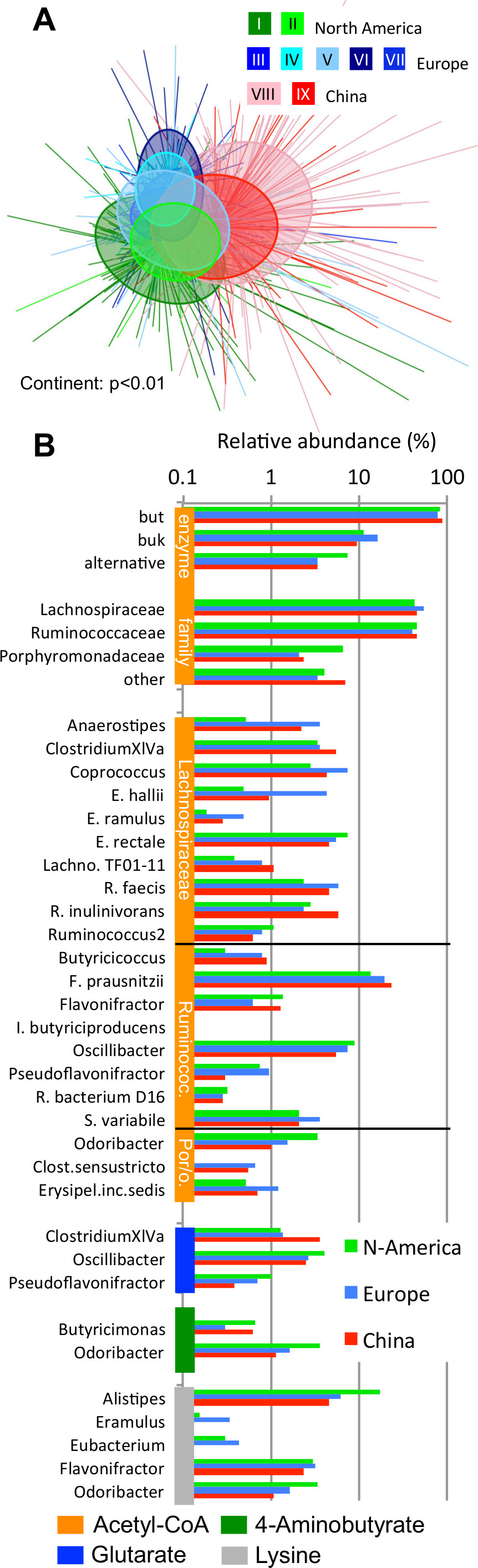

Supplement: FIG S2 [file sys006172157sf2.jpg]

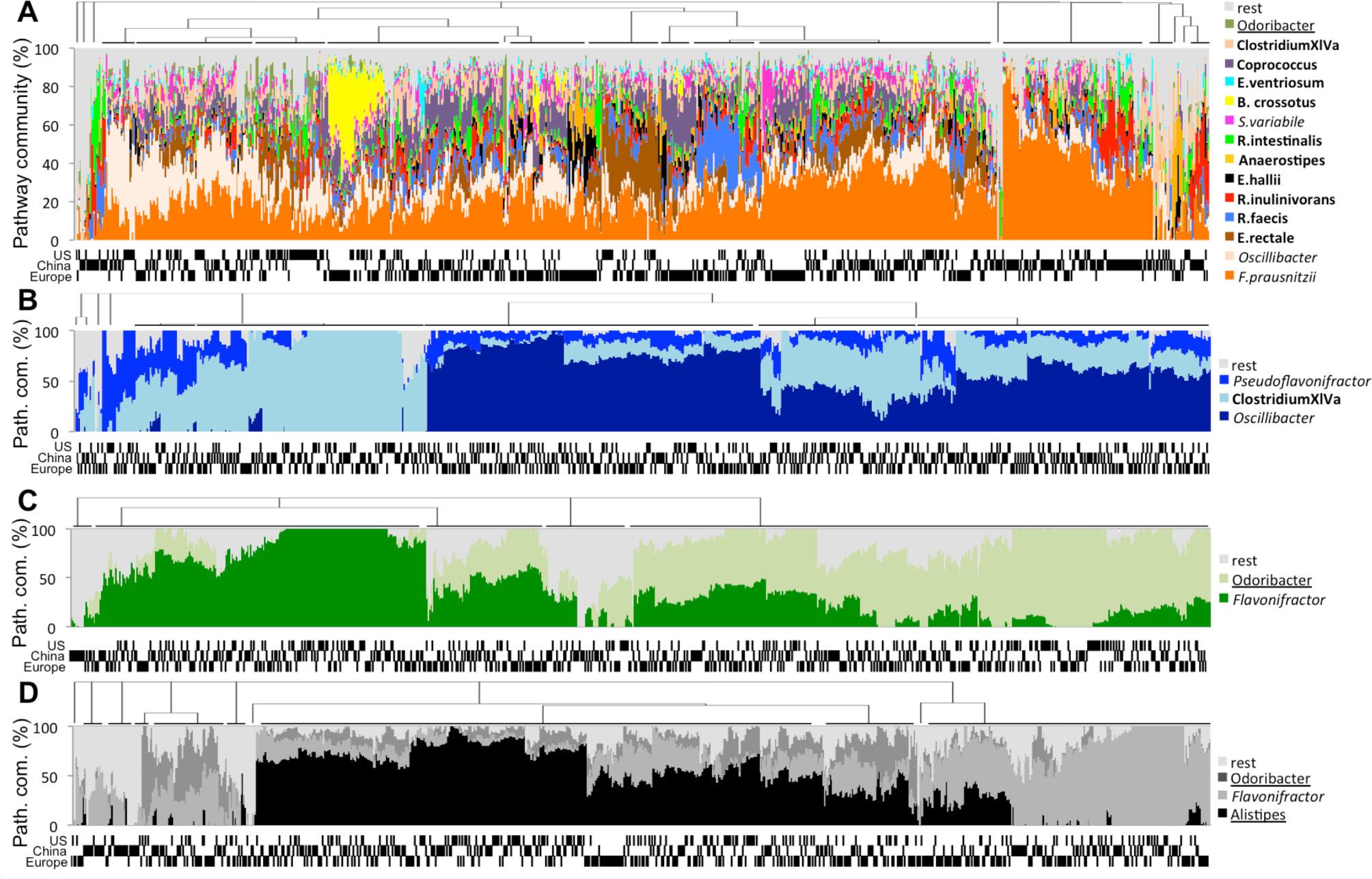

Supplement: FIG S3 [file sys006172157sf3.jpg]

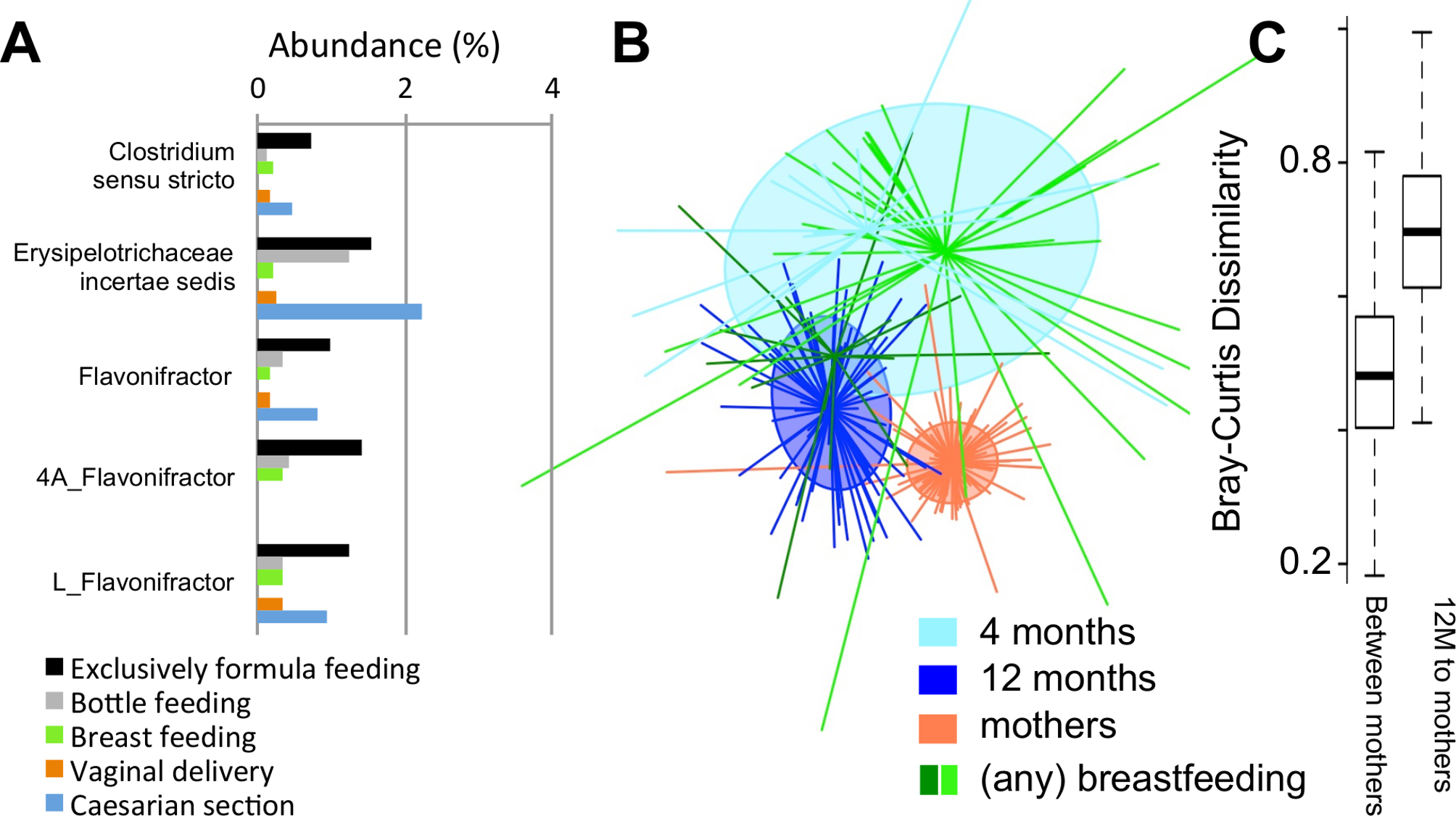

Supplement: FIG S4 [file sys006172157sf4.jpg]

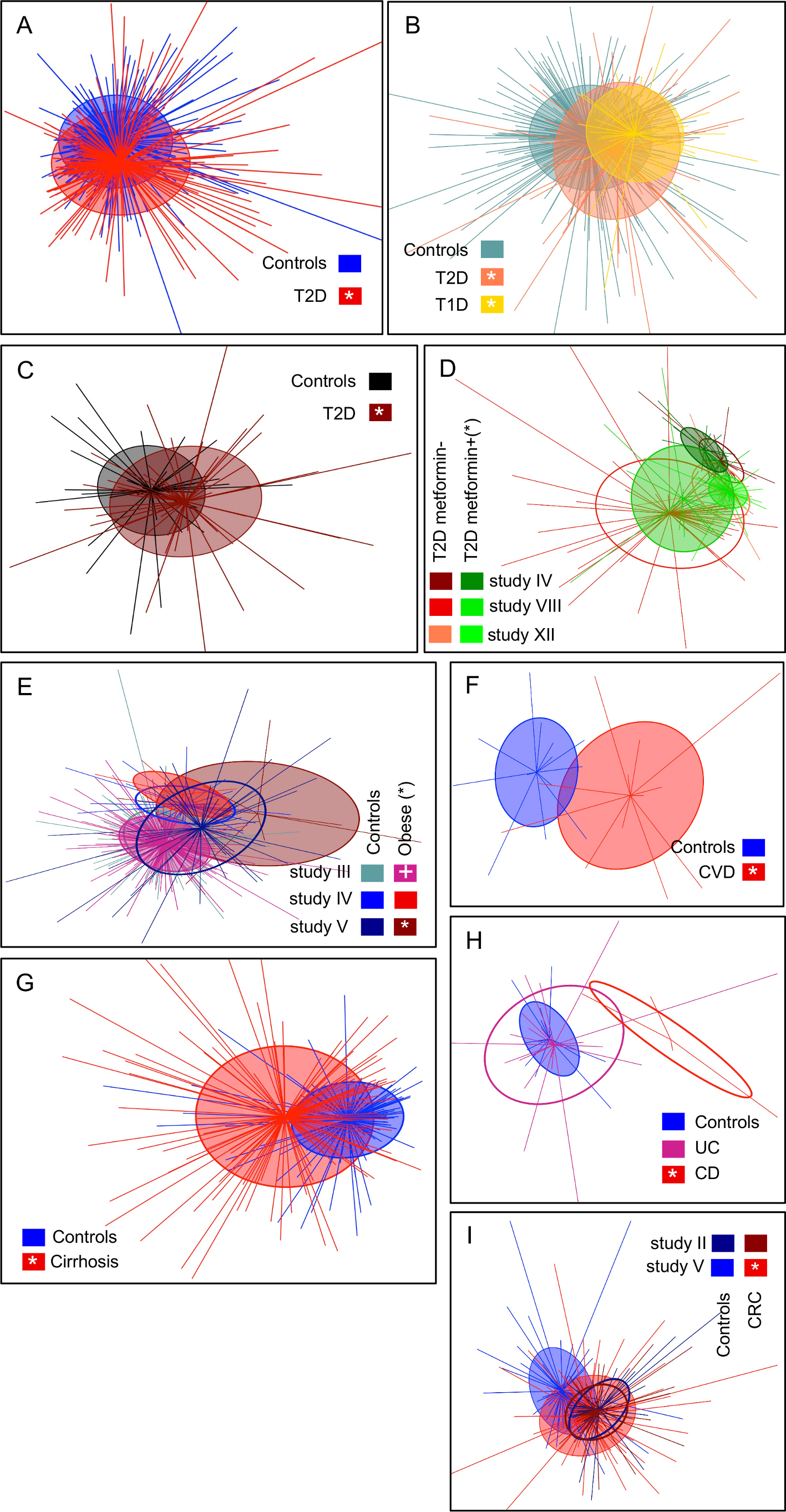

Supplement: FIG S5 [file sys006172157sf5.jpg]

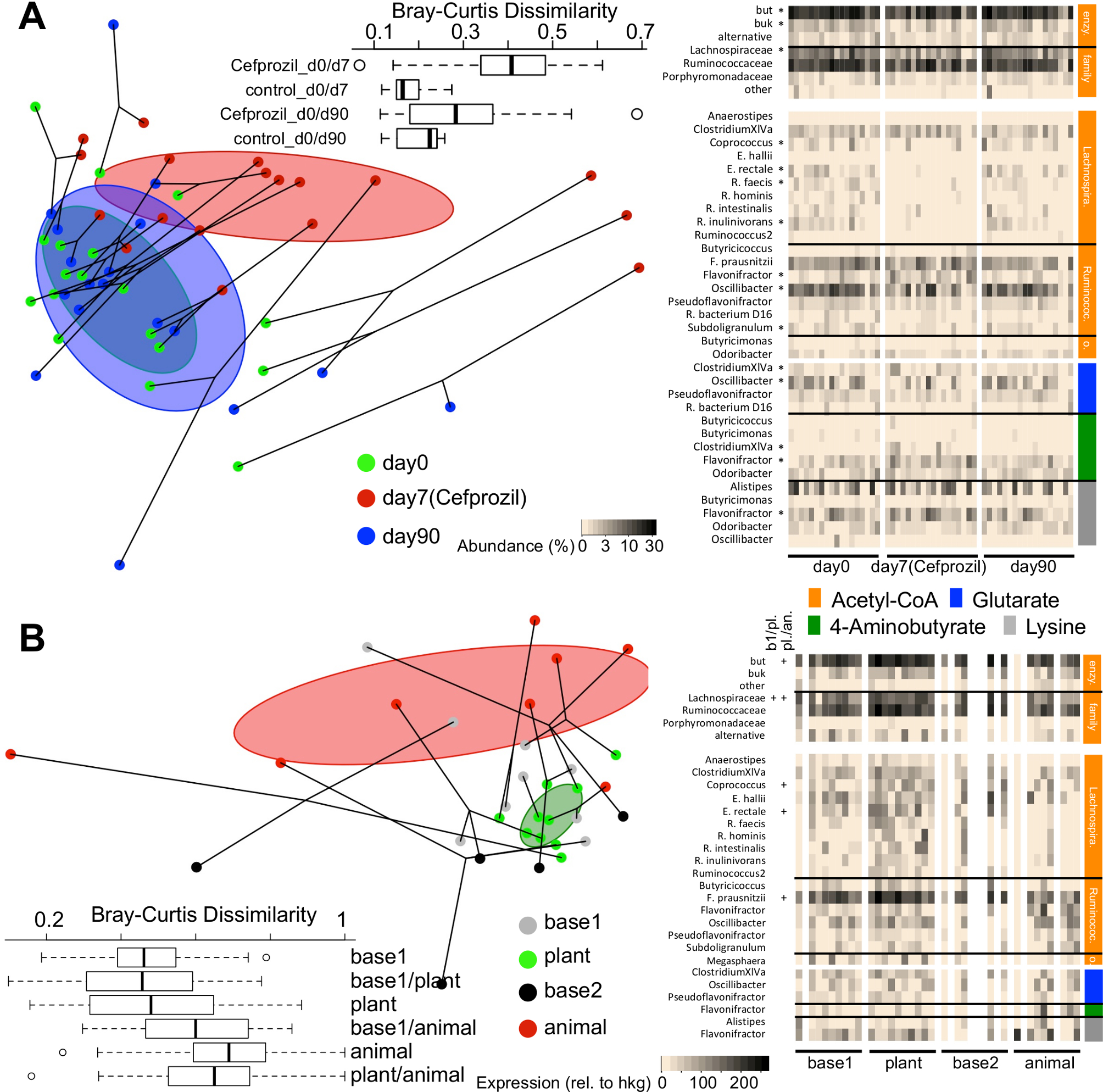

Supplement: FIG S6 [file sys006172157sf6.jpg]

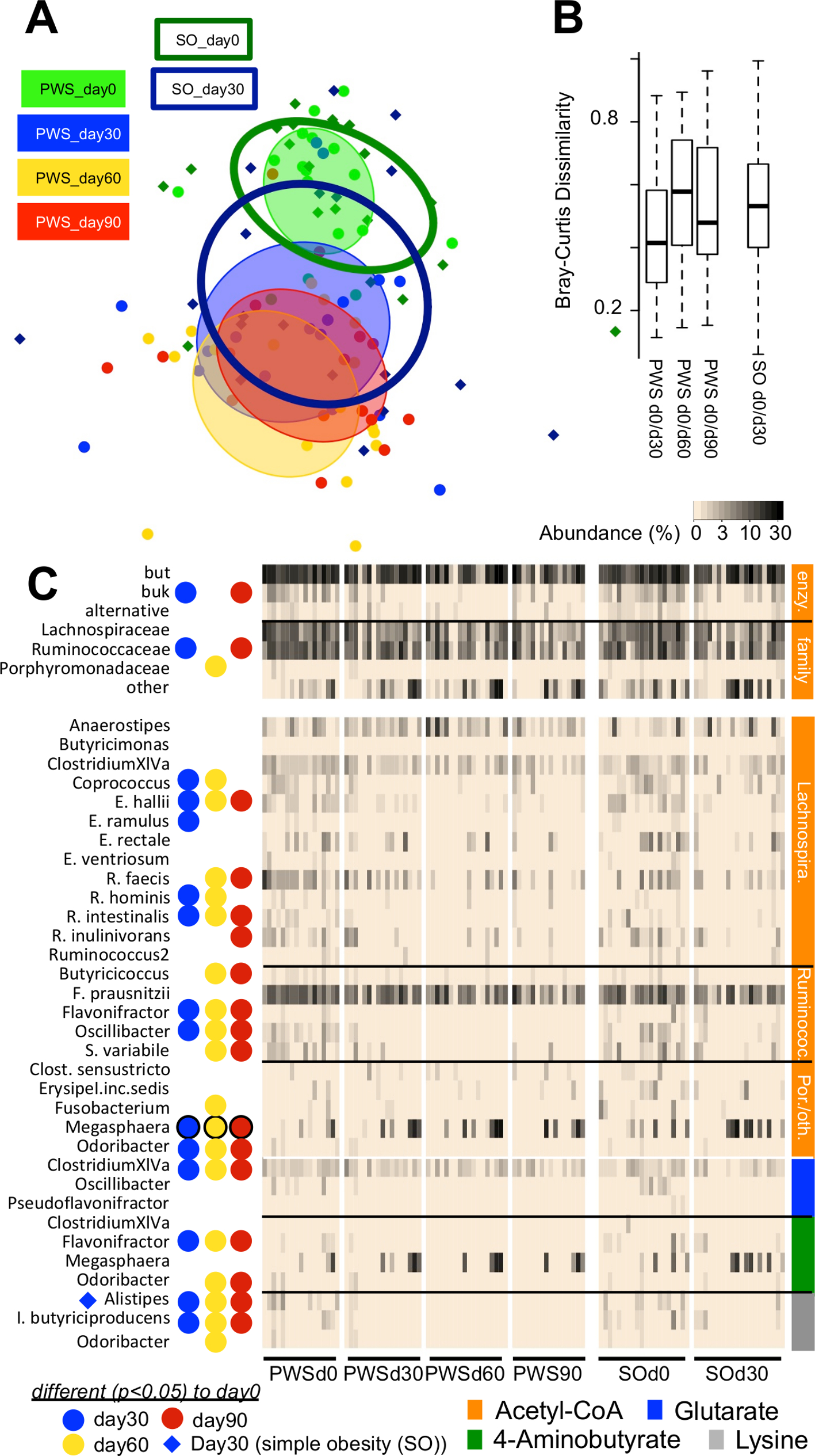

Supplement: FIG S7 [file sys006172157sf7.jpg]

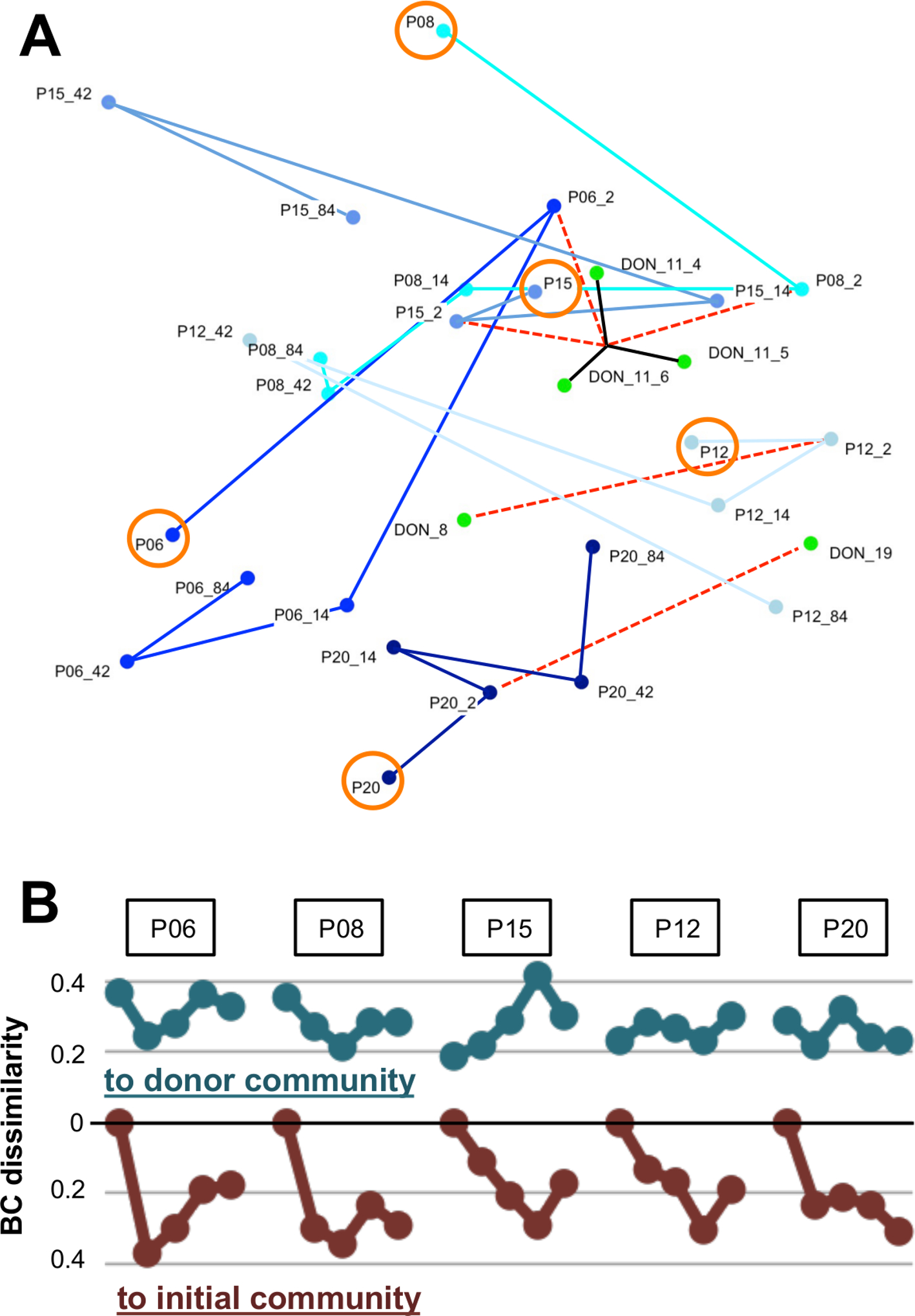

Supplement: FIG S8 [file sys006172157sf8.jpg]
